# A Novel Approach Based on Acceptor–Donor–Acceptor Type Molecules Enhances the Therapeutic Efficacy and Safety of Photothermal Therapy for Osteosarcoma

**DOI:** 10.1002/advs.75482

**Published:** 2026-05-14

**Authors:** Zhijian Jin, Hongru Wang, Yi Yang, Yuhang Xie, Yuan Zhang, Zhiye Du, Yi Huang, Wei Guo, Yang Li, Shidong Wang

**Affiliations:** ^1^ Department of Musculoskeletal Tumor Peking University People s Hospital Beijing China; ^2^ Beijing Key Laboratory of Musculoskeletal Tumor Peking University People s Hospital Beijing China; ^3^ Department of Neurology Liaocheng People's Hospital Liaocheng Shandong China; ^4^ Peking University Health Science Center Beijing China; ^5^ Department of General Surgery the First Medical Center Chinese PLA General Hospital Beijing China

**Keywords:** acceptor–donor–acceptor type molecules, cancer photothermal therapy, nonradiative decay, near‐infrared irradiation, osteosarcoma, photothermal agents

## Abstract

Photothermal therapy based on acceptor–donor–acceptor (A‐D‐A) type molecules is emerging as a powerful clinical treatment strategy, yet its potential application in malignant osteosarcoma has been scarcely investigated. To resolve this, we constructed novel photothermal reagents from A‐D‐A molecules, boosting PCE of A‐D‐A photosensitizers by regulating intramolecular push–pull interactions. Through an optimized push–pull architecture, the IEICO‐F molecule achieved an ideal overlap between its absorption spectrum and the 808 nm NIR laser, along with a high absorption coefficient and an increased nonradiative decay rate constant. Accordingly, following 5‐min 808 nm laser exposure (0.33 W cm^−^^2^), the temperature of IEICO‐F nanoparticles climbed to 63.8°C, exceeding IEICO nanoparticles by 9.2°C. These nanoparticles effectively generated heat to kill osteosarcoma cells and exhibited significant tumor suppression in osteosarcoma models. Importantly, our molecular design also ensures good safety profiles for practical medical application. Therefore, this novel A‐D‐A type molecule‐based photothermal therapy strategy holds promise for widespread clinical use in osteosarcoma treatment and may be extendable to other tumor types in the future.

## Graphical abstract

Jin et al. developed IEICO‐F nanoparticles with enhanced photothermal conversion efficiency via optimized push‐pull effect. Under 808 nm laser, these nanoparticles effectively generated heat to kill osteosarcoma cells and exhibited significant tumor suppression in vivo. Therefore, this novel A‐D‐A type molecule‐based photothermal therapy strategy holds promise for widespread clinical use in osteosarcoma treatment and may be extendable to other tumor types.

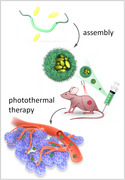

## 1 Introduction

Osteosarcoma represents the prevalent primary bone sarcoma, chiefly affecting children and young adults [1]. Modern therapeutic regimens combine multidrug chemotherapy with surgical resection. However, this strategy remains far from sufficiently effective, as patient survival rates have not improved within the past several decades [2, 3]. Photothermal therapy (PTT) has emerged as an advanced and promising therapeutic strategy for malignant tumor suppression, offering superior intrinsic advantages including potent tumor‐inhibitory capacity, alleviated multidrug resistance, minimized systemic toxicity, and negligible adverse biological side effects [4, 5, 6, 7]. As a favorable noninvasive therapeutic modality, PTT relies heavily on functional photothermal agents (PTAs) to convert exogenous light energy into localized heat, where high‐performance photosensitizers serve as the core functional mediators to guarantee effective tumor suppression and precise photothermal treatment [8]. Under near‐infrared (NIR) light, PTAs turn absorbed photon energy into heat, inducing localized hyperthermia that achieves cancer cell ablation with little surgical trauma or pain [9, 10, 11].

For an effective NIR‐absorbing photosensitizer, a high photothermal conversion efficiency (PCE) is critically important, which not only enables a low PTA dosage but also reduces the required light irradiation intensity during PTT [12, 13, 14]. However, most existing photosensitizers suffer from inadequate PCEs and have to be operated under high‐power NIR irradiation exceeding 1 W·cm^−2^. In clinical treatment, the maximum permissible exposure (MPE) is recognized to be 0.33 W·cm^−2^ (808 nm), which is regarded as a standard safety requirement. At present, the lack of photosensitizers with high PCE has become a major obstacle to clinical application of PTT technology [15, 16, 17].

Desirable PTAs exhibit essential features as follows: 1) high absorption coefficient at the laser wavelength; 2) high PCE; 3) excellent biosafety. In recent years, a large variety of NIR‐absorbing materials, including 2D nanosheets, inorganic nanoparticle, carbon nanotubes, gold nanorods, organic compounds, and conjugated polymers, were developed as photosensitizers [18, 19, 20, 21, 22, 23, 24]. For developing NIR PTAs, acceptor‐donor‐acceptor (A‐D‐A) type organic small molecules show particular advantages among these materials, thanks to their tunable photophysical properties and outstanding biocompatibility [8, 25, 26]. Distinct structural variations in molecular frameworks grant A‐D‐A type organic compounds unique functional advantages over conventional materials. Such tunable chemical architectures empower these tailor‐made molecules to attain excellent molar extinction properties and optimized photothermal conversion efficiencies for versatile bioapplication scenarios [27, 28, 29]. To date, through rational molecular design, the PCE of A‐D‐A type photosensitizers has been significantly enhanced from 27% to over 60%. Despite advances in the PTT performance of A‐D‐A type photosensitizers, the correlation between the chemical structure and PCE remains unclear, making it a great challenge to further enhance the PCEs [30, 31]. As a result, with only a few exceptions, most A‐D‐A type PTAs can barely achieve moderate PTT efficacy under high‐power NIR irradiation (1 W·cm^−2^)—a power level that severely violates medical safety standards—thereby critically constraining their practical application [32]. Thus, it is imperative to explore new ways to enhance the PCE of A‐D‐A molecules, so as to enable their use as PTAs for clinical tumor therapy under standard medical NIR irradiation.

The intramolecular push–pull electronic effect holds great application potential across diverse functional material systems. This classic molecular design strategy has been extensively exploited in organic photovoltaics, memory storage units, light‐emitting elements, nonlinear optical materials, and photothermal agents for biomedical use [33, 34]. Most NIR dyes were developed by means of push‐pull structures to optimize optoelectronic properties such as absorption spectrum, ε, and energy level, so as to improve their performance in PTT. For instance, Li et al. introduced a strong push‐pull system into an aggregation‐induced emission luminogen, and developed a NIR photosensitizer NTT‐BBT [35]. The pronounced charge transfer effect in NTT‐BBT enhanced both the PCE and the generation of reactive oxygen species, contributing to the outstanding performances in PTT and photodynamic therapy. Besides intrinsic optoelectronic properties, molecules with electronic push‐pull system also have unique influence on aggregation structure. Since photosensitizers are typically assembled into nanoparticles (NPs) for applications, the specific properties induced by aggregation represent a key mechanism for boosting their performance. Recently, Xing et al. synthesized a NIR BODIPY dyes with electronic push‐pull system, exhibiting a pounced J‐aggregate with a redshift of absorption spectrum, which demonstrated synergistic therapeutic effects in HepG2 cells and tumor‐bearing mice [11]. Unfortunately, how the intramolecular push‐pull effect governs aggregation in A‐D‐A molecules remains underexplored, limiting further gains in PTA performance. Thus, constructing A‐D‐A‐type molecules with an optimized electronic push‐pull system is strongly needed to enhance the PCE of PTAs. In most tumors, chemotherapy is effective but causes many serious side effects [36, 37]. In recent years, many biomaterials have emerged to treat various diseases, but they all have limitations [38]. By using PTT, we can effectively kill tumors while basically producing no side effects.

In this work, a set of tailored A‐D‐A molecular derivatives were rationally synthesized to modulate intramolecular push–pull electronic interactions. Reasonable regulation of such molecular characteristics effectively elevates the photothermal conversion performance of functional photothermal agents, thereby enabling safe and feasible in vivo PTT implementation under clinical biosafety criteria. The intramolecular push‐pull effect in fluorinated IEICO‐F leads to the optimal overlap between its absorption spectrum and the 808‐nm NIR laser. Besides, IEICO‐F exhibited manifold advantages of high absorption coefficient, compact molecular packing and sufficient non‐radiative decay rate constant, contributing to a high PCE of 65.59%. Under a low‐power NIR irradiation with medical safety (0.33 W·cm^−2^), the IEICO‐F NP with 100 µg·mL^−1^ yield a death rate of over 92% for diverse osteosarcoma cells, and exhibits a complete elimination of tumor bulk. The in vivo experimental results confirmed that the IEICO‐F‐based NPs showed high biosafety, exhibiting low toxicity toward normal tissues. Our findings demonstrate that the manipulation of intramolecular push‐pull effect provides an effective approach to achieve high PCEs in the development of photosensitizers for efficient cancer PTT applications under medical safety standards.

## 2 Results and Discussion

### 2.1 Molecular Design, Characterization, and Photophysical Properties

**FIGURE 1:**
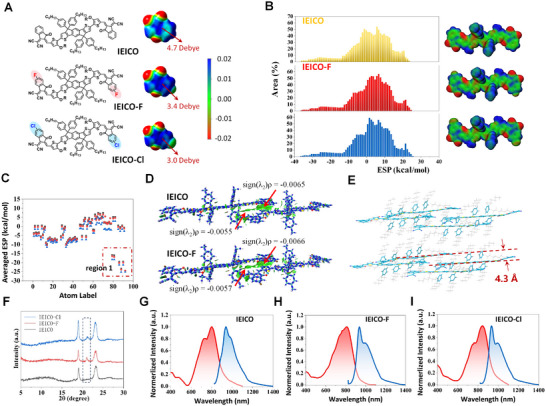
Molecular design, characterization of IEICO, IEICO‐F, and IEICO‐Cl. A) Chemical structures and dipoles of ending groups for IEICO, IEICO‐F, and IEICO‐Cl. B) ESP area distribution of the photosensitizer molecules. C) Averaged ESP values of individual atoms for the photosensitizer molecules (region 1: heteroatoms in the photosensitizers). D) NCI maps of two layers for IEICO and IEICO‐F. The sign(*λ*_2_)*ρ* values are mapped on the RDG isosurfaces. E) Simulation of intermolecular stacking of optimized IEICO‐F. F) XRD pattern of IEICO, IEICO‐F, and IEICO‐Cl. Absorption spectra of G) IEICO, H) IEICO‐F, and I) IEICO‐Cl solutions. ESP, electrostatic potential. NCI, non‐covalent interaction. RDG, reduced density gradient. XRD, X‐ray diffraction.

The compound IEICO is commercially available, whereas the molecules IEICO‐F and IEICO‐Cl are synthesized via the Knoevenagel condensation of the intermediate IE‐CHO with fluorinated E‐F and chlorinated E‐Cl compounds, respectively (Figure S1). The synthetic methods followed the procedures reported in previous works [39, 40]. ^13^C‐NMR spectrum (400 MHz) and the MALDI‐TOF spectrum of IEICO were shown in Figures S2 and S3. Molecular configurations of IEICO‐F and IEICO‐Cl were authenticated through 1H NMR, 13C NMR, and MALDI‐TOF spectral measurements (Figures S4–S9). To analyze the dipole and electrostatic potential (ESP) distribution of the ending groups, we performed density functional theory (DFT) calculations at the B3LYP/6‐31G(d,p) level. The dipoles of E‐H, E‐F, and E‐Cl are 4.7, 3.4, and 3.0 Debye, respectively (Figure 1A). The decreased dipole values imply a more electron‐deficient characteristic of ending groups, which will enhance the intramolecular push‐pull effect in the A‐D‐A molecules. Presented in Figure 1B are surface potential contours of IEICO, IEICO‐F, and IEICO‐Cl, which were calculated atop molecular van der Waals boundaries at a fixed electron density threshold of 0.001 a.u. These A‐D‐A structured compounds delivered obviously asymmetric surface potential features with widely dispersed numerical ranges. The dominant surface coverage fell steadily between 5 and 12 kcal·mol^−^^1^. For more intuitive comparison, mean atomic potential data of the above three molecular samples were summarized in Figure 1C. Carbon sites bonded to halogen substituents in fluorinated and chlorinated derivatives carried much stronger positive potential relative to the original IEICO skeleton. This observable difference reveals that terminal halogen modification can distinctly amplify internal surface potential polarization across the whole molecular framework. This may induce stronger intramolecular push‐pull effects and intermolecular electrostatic interactions.

To demonstrate the influence of intramolecular push‐pull effect and ESP difference on molecular packing, we implemented computational simulations to forecast stacking arrangements of these A‐D‐A‐based compounds under aggregated condensed states. From periodic DFT calculations, Multiwfn generated the non‐covalent interaction (NCI) maps of IEICO and IEICO‐F, as shown in Figure 1D. The color‐filled interaction region indicator (IRI) maps can clearly depict the multiple noncovalent interact regions between two adjacent molecules. Compared to IEICO, the lower value of sign(*λ* ^2^)*ρ* indicates that intermolecular *π*–*π* interaction becomes stronger in the fluorinated IEICO‐F. This indicates that IEICO‐F with stronger push‐pull effects is readily assembled through *π*–*π* stacking interactions. The intermolecular interaction of IEICO‐Cl is lower, as compared to those of IEICO and IEICO‐F, which may be due to the large size of chlorine atom (Figure S10). The molecular stacking modes were obtained by DFT calculation using the PBE functional and DZVP MOLOPT basis set. As shown in Figure 1E, the *π*–*π* stacking distance for IEICO‐F was calculated to be 4.3 Å. The *π*–*π* stacking distances for IEICO and IEICO‐Cl were 4.5 and 4.3 Å, respectively (Figure S11). With enhanced push‐pull structures, IEICO‐F and IEICO‐Cl exhibited compact molecular packing, which may favor the non‐radiative decay, so as to enhance the PCE. To validate the theoretical conclusions, X‐ray diffraction (XRD) was used to assess the intermolecular spacing of the A‐D‐A molecules in their aggregated state. Figure 1F shows the XRD patterns for bulk powder samples of the A‐D‐A photosensitizers. The samples of IEICO, IEICO‐F, and IEICO‐Cl showed diffraction peaks at 23.2°, 23.3°, and 23.3°, respectively. These diffraction peaks originated from the *π*–*π* stacking of A‐D‐A molecules. By analyzing characteristic diffraction signals, the intermolecular layer spacing was calculated to be approximately 3.9 Å for all three A‐D‐A photoactive compounds in condensed solid form. The compact molecular packing can facilitate nonradiative decay, which may contribute to an enhancement in PCE. It is worth noting that, compared to IEICO, the halogenated IEICO‐F and IEICO‐Cl samples exhibit distinctive diffraction peaks at 21.1°, indicating the additional architecture of ordered arrangement for A‐D‐A molecules. As revealed by the DFT calculations, the halogenated A‐D‐A photosensitizers with stronger push‐pull architectures exhibited strengthened intermolecular interaction, which may be responsible for the enhanced ordering of molecular arrangement. The experimental data accorded with the results from the theoretical study, confirming that the modulation of intramolecular push‐pull effect could optimize the packing architecture of photosensitizers in the aggregate state.

The construction of intermolecular push‐pull architecture is one of the most widely used approach to modulate optical properties of organic compounds. Thus, the absorption and fluorescence spectra of the A‐D‐A photosensitizers were measured. As shown in Figure 1G–I, the absorption peaks of IEICO, IEICO‐F, and IEICO‐Cl solutions were located at 800, 811, and 843 nm, respectively. These peaks originate from *π*–π* electronic transitions of the conjugated framework, reflecting an enhanced push‐pull effect in the halogenated A‐D‐A molecules. The fluorescence spectra of all photosensitizer solutions show peaks at around 938 nm. The photosensitizer IEICO‐F exhibits the best overlap with the 808‐nm medial laser, thereby benefiting an enhancement in PCE. These findings validate the effectiveness of our material design strategy. By manipulating the intramolecular push‐pull effect, the absorption spectra of the A‐D‐A photosensitizers can be finely tuned, thus achieving a better match between the PTA absorption and the medical laser wavelength.

**FIGURE 2:**
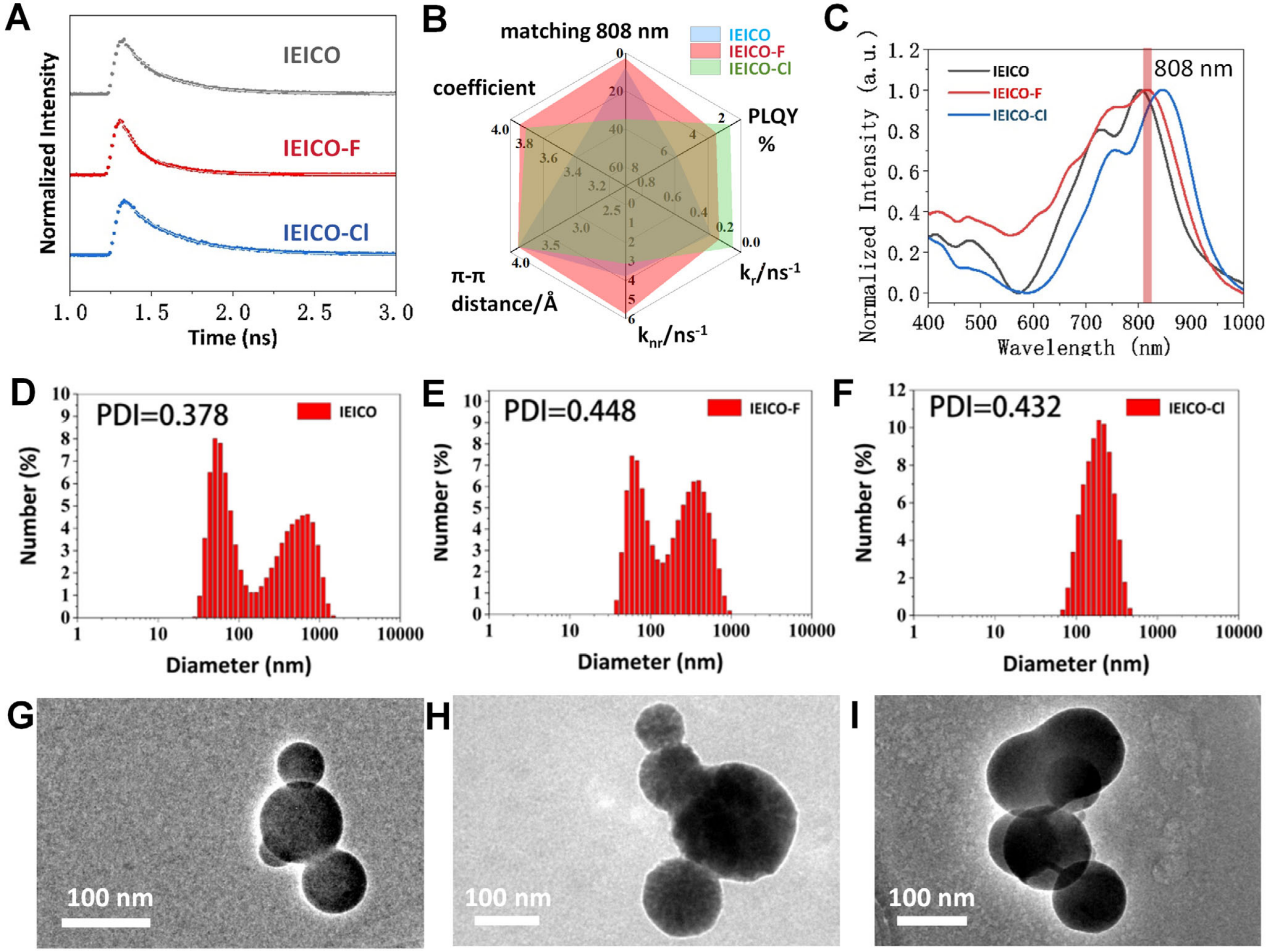
Photophysical properties of IEICO, IEICO‐F, and IEICO‐Cl. A) Transient PL attenuation spectra of A‐D‐A photosensitizers as solid films. B) Radar chart of property parameters for the photosensitizers. C) Absorption spectra of NPs in aqueous solutions. D) IEICO, E) IEICO‐F, and F) IEICO‐Cl NPs. Transmission electron microscopy images of G) IEICO, H) IEICO‐F, and I) IEICO‐Cl NPs. Scale bar: 100 nm. NPs, nanoparticles. PDI, polydispersity index. PTT, photothermal therapy.

To verify the theoretical predictions, lifetime of fluorescence (τ) for the A‐D‐A type molecules was measured to investigate the impact of push‐pull effect on nonradiative decay. The photoluminescence quantum yields (PLQYs) of IEICO, IEICO‐F, and IEICO‐Cl were determined to be 5.40%, 2.69%, and 1.70%, respectively (Figure S12). A low PLQY implies efficient channels for non‐radiative decay, which is favorable for achieving high PCE. As shown in Figure 2A, Time‐resolved luminescence attenuation curves of these nanoparticle samples in water were experimentally tested. The recorded radiative decay durations reached 0.25 ns for IEICO, 0.17 ns for IEICO‐F, and 0.31 ns for IEICO‐Cl in sequence. Based on the *τ* values, the radiative decay rate constants (*k*_r_) are calculated to be 0.22, 0.16, and 0.055 ns^−1^ for IEICO, IEICO‐F, and IEICO‐Cl, respectively. By combining *k*_r_ and PLQY, the nonradiative decay rate constants (*k*_nr_) of IEICO, IEICO‐F, and IEICO‐Cl can be calculated to be 3.85, 5.79, and 3.18 ns^−1^, respectively. The results suggest that the construction of strong push‐pull structure can greatly enhance the *k*_nr_ of the A‐D‐A molecules, which provides a new channel for PCE enhancement. Afterward, the mole extinction coefficients of IEICO, IEICO‐F, and IEICO‐Cl in tetrahydrofuran solutions were determined to be 3.87 × 10^3^, 4.92 × 10^3^, and 3.49 × 10^3^ L·mol^−1^·cm^−1^ (Figure S13). As depicted in Figure 2B, this radar chart visually contrasts key optical performance parameters across the prepared A‐D‐A molecular derivatives. The IEICO‐F demonstrates a well‐balanced combination of photophysical properties: optimal spectral overlap with the 808‐nm medical laser, a high absorption coefficient, and a large nonradiative rate constant—all of which are recognized as critical factors for achieving high PCE values. Thus, it is reasonably expected that IEICO‐F can achieve a high PCE in NPs, enabling PTT applications under medical safety standards.

Next, the standard coprecipitation protocol, with Pluronic F‐127 encapsulating organic moieties, was employed to prepare the three photosensitizers as nanoparticles. As described in the experimental section, the encapsulation yields of NPs are over 70%.

The zeta potential values of the IEICO, IEICO‐F, and IEICO‐Cl NPs were measured to be −29.8, −32.4, and −28.3 mV, respectively. The results suggested that the NPs were well dispersed in aqueous solution and had a negative charge on their surfaces (Figure S14). As shown in Figure 2C, the absorption spectra of NPs keep almost unchanged as compared to those of the photosensitizer solutions. The results demonstrate again that the IEICO‐F NP displays the best spectral overlap with the 808‐nm medical laser. Dynamic light scattering (DLS) revealed broad NP size distributions, with average diameters of 65, 76, and 197 nm for IEICO, IEICO‐F, and IEICO‐Cl NPs, respectively (Figure 2D–F). However, the polydispersity index (PDI) values of the NP sizes are 0.378, 0.448, and 0.432 for the IEICO, IEICO‐F, and IEICO‐Cl NPs. These values are indicative of a non‐uniform size distribution, primarily due to the aggregation of NPs. In the DLS results for IEICO and IEICO‐F NPs, the peaks observed in the hundred‐nanometer range can also be attributed to NP aggregation. Transmission electron microscopy was used to investigate NP morphology. As shown in Figure 2G–I, all NPs displayed well‐formed spherical structures with average diameters around 100 nm. These sizes are sufficient for passive tumor accumulation via the enhanced permeability and retention (EPR) effect. The mass extinction coefficients (*ε*) of IEICO, IEICO‐F, and IEICO‐Cl NPs were 2.87, 4.78, and 3.10 L·mg^−1^·cm^−1^, respectively (Figure S15). The high *ε* value of the IEICO‐F NP can ensure a sufficient NIR absorption, which is essential for achieving high PCE in the PTA. These findings validate the effectiveness of our material design strategy. By tailoring the push‐pull architecture, the absorption spectra of the A‐D‐A photosensitizers can be precisely adjusted to better match the medical laser wavelength, thereby enhancing PTA performance. The fluorescence emission of IEICO, IEICO‐F, and IEICO‐Cl NPs in aqueous dispersion is shown in Figure S16.

### 2.2 Photothermal Properties

**FIGURE 3:**
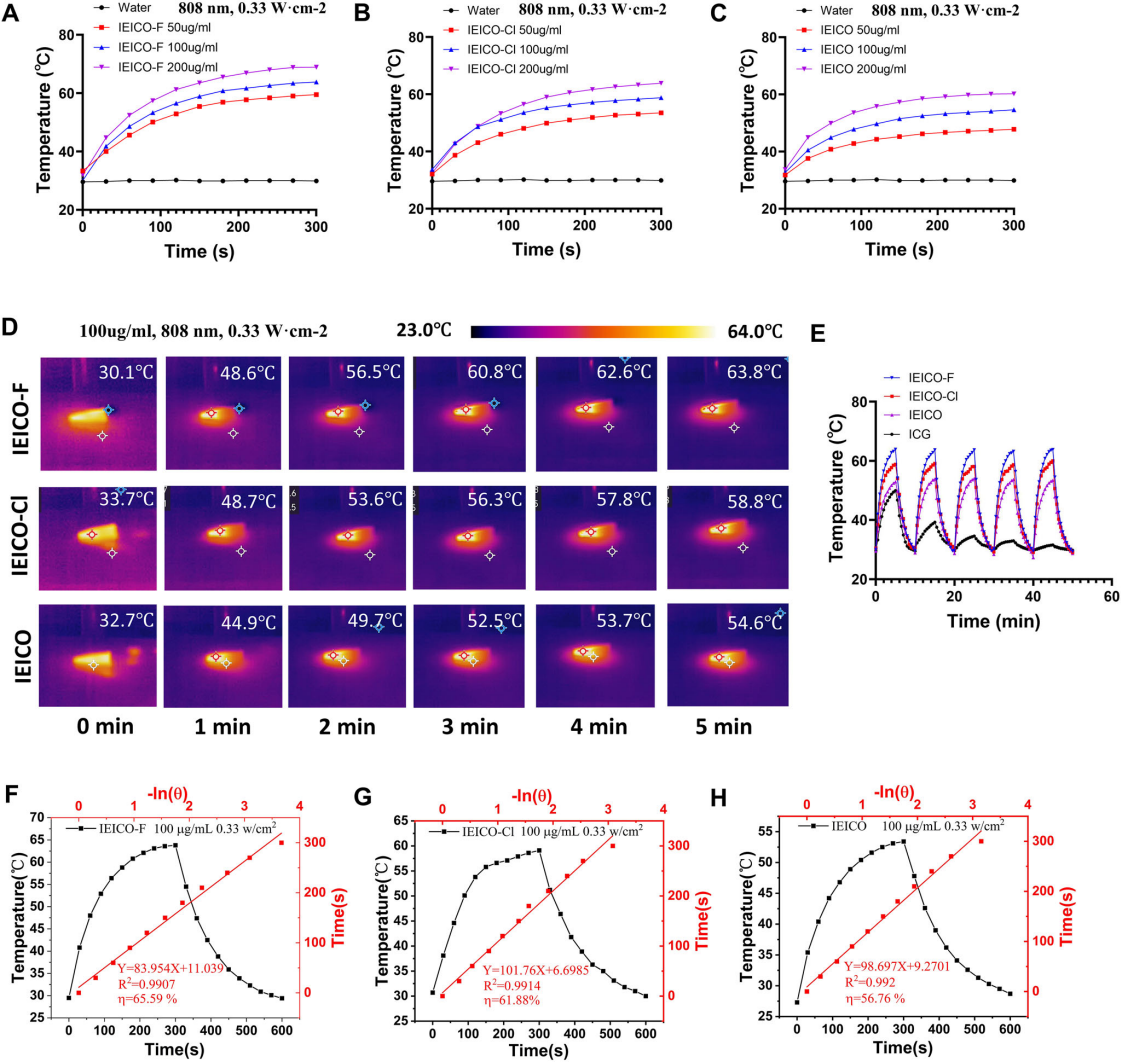
Photothermal properties of IEICO‐F, IEICO‐Cl, and IEICO NPs. Photothermal temperature change curves of A) IEICO‐F, B) IEICO‐Cl, and C) IEICO NPs at different concentrations upon laser irradiation (808 nm, 0.33 W·cm^−2^). D) The infrared thermal images of IEICO‐F, IEICO‐Cl, and IEICO NPs (100 ug·mL^−1^) at different times during 5 min of laser irradiation (808 nm, 0.33 W·cm^−2^). E) Thermostability of IEICO‐F, IEICO‐Cl, IEICO, and ICG NPs (100 ug·mL^−1^) under laser irradiation (808 nm, 0.33 W·cm^−2^) for five cycles. Heating and cooling curves of 100 ug·mL^−1^ F) IEICO‐F, G) IEICO‐Cl, and H) IEICO NPs under laser irradiation (808 nm, 0.33 W·cm^−2^) for 5 min, and linear time data vs −ln(𝜃) from the cooling period. NPs, nanoparticles.

As shown in Figure 3A–C, the photothermal properties of IEICO‐F, IEICO‐Cl, and IEICO NPs aqueous dispersions at different concentrations under low‐power near‐infrared (NIR) laser irradiation were investigated by recording the temperature changes. Under continuous low‐power NIR laser irradiation (808 nm, 0.33 W·cm^−^^2^, 5 min), the temperature of all NPs aqueous dispersions increased rapidly at various concentrations, whereas pure water showed no temperature rise. Notably, at an IEICO‐F NPs concentration of 100 µg·mL^−^^1^, the dispersion temperature reached a high plateau of 63.8°C, achieving a temperature increase (ΔT) of 33.7°C. Given the threshold temperature for photothermal therapy (43.0°C) and the average body temperature of anesthetized mice (35°C), this ΔT of 33.7°C is sufficient to exceed the required 8°C temperature rise, enabling the complete eradication of cancer cells in live mice. A ΔT of 26.2°C was attainable even with IEICO‐F NPs at a low concentration of 50 µg·mL^−^^1^. These results indicate that IEICO‐F NPs can serve as excellent photothermal agents (PTAs). Figure 3D showed the infrared thermal images of IEICO‐F, IEICO‐Cl, and IEICO NPs (100 µg·mL^−^^1^) at different time points during 5 min of laser irradiation (808 nm, 0.33 W·cm^−^^2^). We next evaluated the photothermal stability of the three NPs using five successive on/off cycles of the 808 nm laser (Figure 3E). According to literature reports [40], the photothermal stability of the most commonly used commercial dye, indocyanine green (ICG), decreases significantly, with its temperature dropping to half of the initial value by the second cycle and continuing to decline. By contrast, our synthesized NPs showed no temperature drop after five cycles, demonstrating photothermal stability far superior to ICG. The photothermal conversion efficiencies (PCEs) of IEICO‐F, IEICO‐Cl, and IEICO NPs (100 µg·mL^−^^1^), calculated using the reported method, were determined to be 65.59%, 61.88%, and 56.76%, respectively (Figure 3F–H). The PCE of IEICO‐F NPs was higher than those of IEICO‐Cl and IEICO NPs. The superior PCE of IEICO‐F NPs over IEICO‐Cl and IEICO NPs can be primarily attributed to two factors: 1) the well‐matched absorption band of IEICO‐F NPs with the 808 nm NIR laser; and 2) high non‐radiative decay. These results demonstrate the feasibility of incorporating fluorine to enhance the PCE of NPs, opening new avenues for developing efficient PTAs for PTT applications within medical safety standards.

### 2.3 Cellular Experiments

**FIGURE 4:**
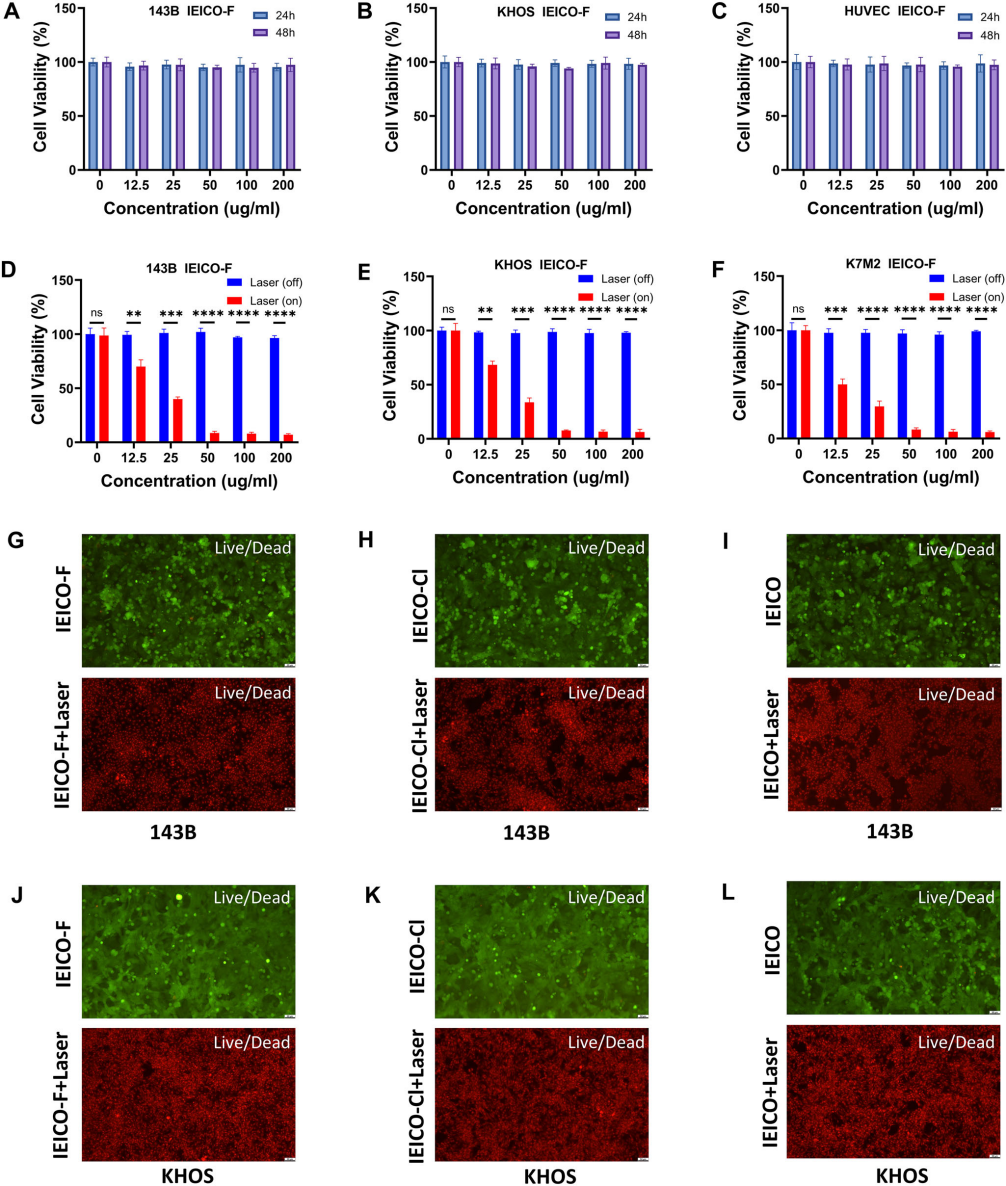
The good biocompatibility and phototherapeutic effect of NPs at the cellular level. Viabilities of IEICO‐F NPs against cancerous A) 143B, B) KHOS, and noncancerous C) HUVEC cells after 24 and 48 h of incubation at different concentrations. Viabilities of cancerous D) 143B, E) KHOS, and F) K7M2 cells incubated with IEICO‐F NPs with or without laser irradiation (808 nm, 0.33 W·cm^−2^, 5 min). G–I) Fluorescence images of calcein AM (green) and propidium iodide (red) co‐stained 143B cells after different treatments. J–L) Fluorescence images of calcein AM (green) and propidium iodide (red) co‐stained KHOS cells after different treatments. NIR light irradiation treatment was conducted for 5 min after cells were incubated with different NPs (100 ug·mL^−1^). Data are presented as the mean ± SD, *n* = 3. *p < 0.05, **p < 0.01, ***p < 0.001, ****p < 0.0001, ns, no significance. Scale bar: 50 µm. NPs, nanoparticles.

Using the CCK‐8 cytotoxicity assay, we evaluated NP biocompatibility. After 24 and 48 h of co‐incubation with IEICO‐F NPs (200 µg·mL^−^^1^), the viability of cancerous 143B cells, KHOS cells, and normal HUVEC cells remained above 95%, indicating good biosafety of IEICO‐F NPs (Figure 4A–C). The cytotoxicity of IEICO‐Cl and IEICO NPs was also assessed, and the cell viability data were similar to those of IEICO‐F NPs (Figure S17A–F). Such findings indicate favorable biosafety and negligible toxicity for all nanoparticles. As shown in Figure 4D, following 5 min of 808 nm laser irradiation (0.33 W·cm^−^^2^), cell viability dropped sharply with rising IEICO‐F NP concentrations. Notably, at 50 µg·mL^−^^1^ IEICO‐F NPs, the mortality rate of 143B cells exceeded 92%. To test universality, we evaluated IEICO‐F NP PTT efficacy in other cancer cell lines; KHOS and K7M2 cell viability results resembled those for 143B cells (Figure 4E,F). The PTT efficacy of IEICO‐Cl and IEICO NPs was also evaluated; cell viability also decreased significantly with increasing NP concentration, but the tumor cell‐killing effect at the same concentration was not as potent as that of IEICO‐F NPs (Figure S17G–L).

To further confirm NP‐mediated cancer cell killing, live/dead staining was performed. Figure 4G–L shows fluorescence images of co‐stained 143B and KHOS cells from different treatment groups, using calcein‐AM (green, live cells) and propidium iodide (red, dead cells). Prior to staining, cells were incubated with various NPs (100 µg·mL^−^^1^) for 24 h. Cells incubated with NPs alone remained alive, displaying green fluorescence. Strikingly, upon 808 nm laser irradiation, cells incubated with NPs showed bright red fluorescence, indicating excellent PTT‐based killing efficiency. 143B and KHOS cells treated with IEICO‐F NPs under 808 nm irradiation showed entire red fluorescence areas, indicating complete killing of cancer cells. These results indicate that IEICO‐F NPs combined with NIR irradiation possess excellent efficacy in killing cancer cells.

Concerning the in vitro antitumor mechanisms of photothermal nanoplatforms, ample documented evidence supports the following pathways: Photothermal therapy leverages robust optical absorption and exceptional PCE of such agents to harvest photon energy and rapidly convert it into thermal energy, raising focal temperatures within tumor lesions. This incites cellular membrane disruption and protein denaturation in malignant cells while leaving healthy tissues unharmed. The fundamental principle of PTT lies in localized hyperthermia that triggers multifaceted alterations in neoplastic cells. Specifically, heat generated by PTT stimulates the liberation of antigens, proinflammatory mediators, and immunogenic intracellular components from apoptotic tumor cells, thereby potentiating antitumor immune activation [41, 42].

### 2.4 In Vivo Photothermal Imaging and PTT Using IEICO‐F NPs

**FIGURE 5:**
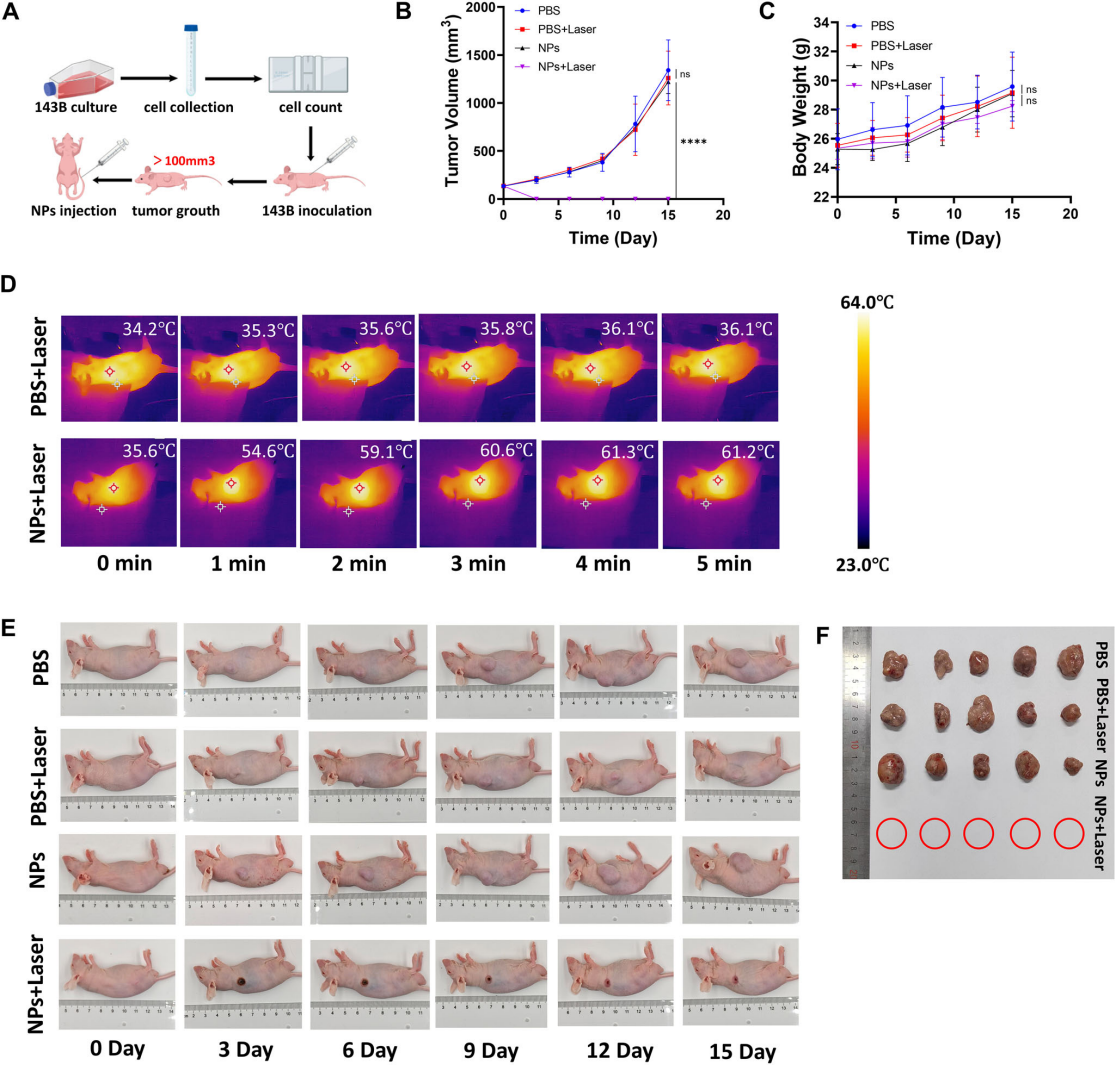
In vivo photothermal imaging and PTT using IEICO‐F NPs. A) Schematic illustration for the in vivo phototherapy. B) Tumor volume changes for various treatment groups. C) Changes in body weights of 143B tumor‐bearing mice after different treatments. D) Infrared thermal images and heating temperatures (at tumor sites) of 143B tumor‐bearing mice during continuous NIR irradiation at 24 h post‐injection of PBS and IEICO‐F NPs, respectively. E) Photographs of different mouse groups during the 15‐day treatment. F) Tumor images harvested after different treatments. Data are presented as the mean ± SD, *n* = 5. *p < 0.05, **p < 0.01, ***p < 0.001, ****p < 0.0001, ns, no significance. PTT, photothermal therapy. NPs, nanoparticles. NIR, near‐infrared.

Based on the high PCE and good biosafety of IEICO‐F NPs, we evaluated their in vivo photothermal therapy efficiency using a mouse model bearing 143B tumors, under 808 nm laser irradiation (Figure 5A). When the tumor volume exceeded 100 mm^3^, mice bearing 143B tumors were randomly divided into four groups (PBS, PBS + Laser, IEICO‐F NPs, IEICO‐F NPs + Laser). Experimental mice were intravenously injected with IEICO‐F NPs (100 µL, 200 µg·mL^−^^1^) via the tail vein. At 24 h post‐injection, the tumor site was exposed to an 808 nm laser (0.33 W·cm^−^^2^) for 5 min, and tumor temperature was monitored using an infrared thermal camera. As shown in Figure 5D, after IEICO‐F NP treatment plus 5 min of 808 nm irradiation (0.33 W·cm^−^^2^), the tumor surface temperature reached 61.2°C. This temperature is sufficient to cause irreversible damage to tumor cells. In contrast, tumors treated with PBS alone followed by laser irradiation showed only a minimal temperature increase of approximately 2°C. Beyond revealing the substantial in vivo photothermal effect of IEICO‐F NPs, this imaging performance provides a convenient means for spatiotemporal in situ assessment in PTT applications. Thereafter, tumor volumes and mouse body weights were monitored every three days to evaluate the antitumor efficacy of IEICO‐F NP‐mediated PTT. The results showed that tumors in the IEICO‐F NPs + Laser group were almost completely disappeared by day 3 (2 days after laser irradiation), and no regrowth or recurrence was observed during subsequent monitoring. This indicates that IEICO‐F NPs possess excellent in vivo photothermal therapeutic efficacy under NIR laser irradiation. However, tumors in the other three groups gradually increased in size over the 15‐day observation period, with no significant differences in tumor volume among them (Figure 5B), suggesting that neither laser irradiation nor IEICO‐F NPs alone affected tumor growth in mice. Additionally, we noted a slight weight gain of 2–3 grams in all four groups of mice, suggesting that IEICO‐F NPs did not negatively impact the rodents' ability to utilize nutrients (Figure 5C). The body weight changes of each mouse were recorded in Table S1. We photographed the external appearance of the tumors in the mice. In the IEICO‐F NPs + Laser group, the skin at the tumor site gradually healed after tumor regression during the 15‐day treatment period (Figure 5E). All experimental mice were sacrificed after 15 days, and tumors from each group were collected and photographed, further confirming the complete disappearance of tumors in the IEICO‐F NPs + Laser group (Figure 5F). These observations demonstrate that IEICO‐F NPs exhibit excellent PTT efficacy under NIR irradiation and fully comply with medical safety standards.

**FIGURE 6:**
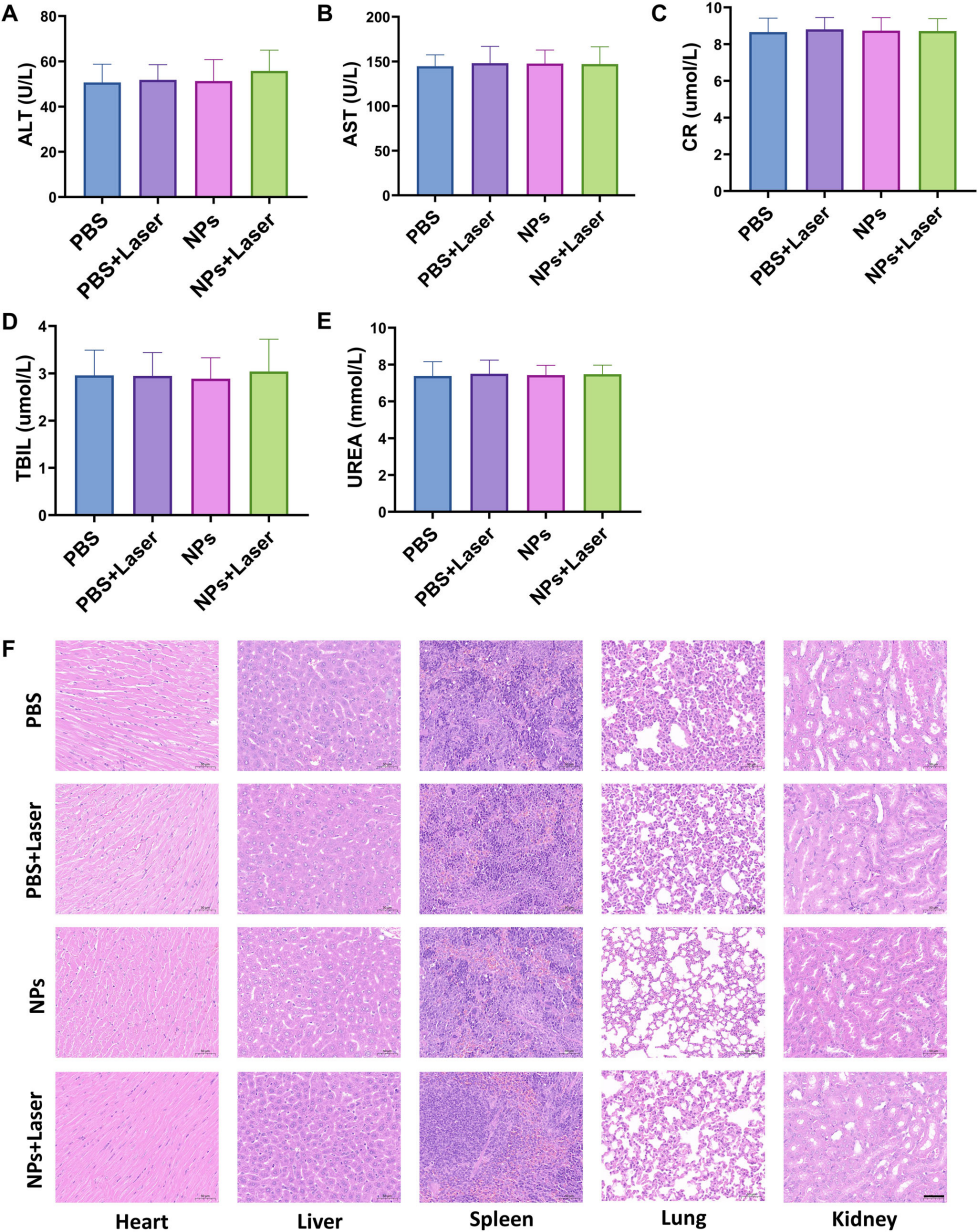
Liver and kidney function parameters as well as H&E staining of mice from different treatment groups. A) ALT, B) AST, C) CR, D) TBIL, E) UREA data of the mice intravenously injected with the IEICO‐F NPs or PBS at 24 h post‐injection. F) Histological analysis of H&E‐stained slices of the main organs of 143B tumor‐bearing mice after various treatments for 15 days. Data are presented as the mean ± SD, *n* = 5. Scale bar: 50 µm. NPs, nanoparticles.

PTA biosafety is essential for biomedical use. We accordingly assessed the biosafety and potential side effects of this PTT regimen. At 24 h post intravenous injection of IEICO‐F NPs or PBS, mouse serum was collected for hepatic and renal function assays (ALT, AST, CR, TBIL, UREA). Notably, no significant differences in these biochemical markers were observed between the IEICO‐F NPs and PBS groups (Figure 6A–E). Fifteen days later, key visceral tissues (heart, liver, spleen, lung, and kidney) collected for histological staining. Organ tissue morphology appeared normal across all groups, with no evident damage (Figure 6F). Collectively, IEICO‐F NPs exhibit favorable in vivo biosafety and potent PTT efficacy under NIR laser irradiation.

## 3 Conclusion

Over the past three decades, osteosarcoma treatment has seen no significant breakthroughs. As a malignant tumor, osteosarcoma typically presents with poorly‐defined margins and is highly prone to relapse or metastasis after surgery. Therefore, we leveraged the ability of nanoparticles to effectively accumulate at tumor sites, enabling photothermal therapy that targets tumors even when they are not visually detectable, thereby achieving potent tumor eradication. Consequently, we tailored the intramolecular push‐pull architecture to enhance the PCE of A‐D‐A photosensitizers, so as to develop high‐performance PTAs for PTT applications under medical safety standards. Through fluorination and chlorination at the ending group, the intramolecular push‐pull effect was strengthened from IEICO to IEICO‐Cl. DFT calculations revealed that a compact molecular packing could be achieved for A‐D‐A molecules with strong push‐pull effect in the aggregation state, as evidenced by XRD results. In particular, IEICO‐F exhibit the best overlap between its absorption spectrum and the 808‐nm NIR laser, which is crucial for PTT applications. Moreover, the photosensitizer IEICO‐F showed an optimal combination of high absorption coefficient and enhanced non‐radiative decay rate constant, leading to a high PCE of 65.59% in the corresponding NP.

In conclusion, IEICO‐2F nanoparticles effectively inhibited osteosarcoma growth and achieved complete tumor ablation under low‐power near‐infrared laser irradiation at 0.33 W·cm^−^^2^. H&E staining results showed that IEICO‐F nanoparticles caused no damage to normal organs/tissues, reaffirming the excellent biosafety of this photothermal agent. Therefore, it holds promise for future osteosarcoma treatment, particularly as a palliative option for advanced, inoperable cases.

## 4 Experimental Section

### 4.1 Instruments and Measurements

Proton nuclear magnetic resonance data were acquired using a Bruker AVANCE III 400 MHz device in chloroform solvent, with residual solvent signal at 7.26 ppm as the internal standard. Mass spectrometric detection was performed on a Bruker Microflex MALDI‐TOF instrument under positive reflector conditions, where 2,5‐dihydroxybenzoic acid (DHB) acted as the ionization matrix. Ultraviolet–visible absorption profiles were detected with a Shimadzu UV‐3600 spectrophotometer, while X‐ray diffraction signals were scanned by a FRINGE CLASS diffractometer. Steady‐state fluorescence signals were collected via an Edinburgh FLS980 spectroscopic analyzer. Dynamic light scattering coupled with a Malvern Zetasizer Nano ZS90 was applied to determine zeta potential and hydrated particle size. Microstructural features and granular morphologies were visualized with a JEOL JEM‐2100 electron microscope. Thermal variation of nanoparticles was tracked by a HIKMICRO infrared camera and further processed with IR Flash thermal analysis software. An 808 nm near‐infrared laser emitter (CNI Laser, MDL‐XD‐808‐5 W) was adopted for photothermal and photodynamic tests. Cell viability absorbance in CCK‐8 assays was quantified on a BioTek microplate detector, and intracellular fluorescent imaging was completed utilizing an Olympus CKX53 inverted microscope.

### 4.2 Synthesis of IEICO‐F/Cl and Structural Characterizations of Materials

Compound 1 (415.2 mg, 0.3 mmol) and compound 2 (1 mmol) were dissolved in anhydrous chloroform (30 mL) in a dry 100 mL two‐necked round‐bottom flask under an argon atmosphere. Pyridine (2 mL) was added, and the mixture was heated under reflux at 65°C for 3.5 h. After cooling to the room temperature, the mixture was poured into methanol and filtrated. The residue was purified by column chromatography on silica gel using chloroform as the eluent to afford compound IEICO‐2F (364.2 mg, 68.5% yield) and IEICO‐2Cl (368.8 mg, 68.1% yield).

IEICO‐2F 1H NMR (400 MHz, Chloroform‐d): δ 8.76–8.67 (m, 2H), 8.37 (dd, J = 9.0, 2.1 Hz, 2H), 7.89 (dd, J = 8.3, 5.1 Hz, 2H), 7.62 (d, J = 2.4 Hz, 2H), 7.54–7.46 (m, 4H), 7.38 (ddd, J = 10.3, 5.2, 2.3 Hz, 2H), 7.21 (d, J = 8.0 Hz, 8H), 7.13 (d, J = 8.0 Hz, 8H), 4.13 (s, 4H), 2.61 (t, J = 7.8 Hz, 8H), 1.88 (p, J = 6.0 Hz, 2H), 1.58 (d, J = 34.2 Hz, 16H), 1.41–1.28 (m, 32H), 1.03–0.87 (m, 24H).

IEICO‐2Cl 1H NMR (400 MHz, Chloroform‐d) δ δ 8.73 (m, 2H), 8.67–8.59 (dd, J = 9.0, 2.1 Hz, 2H), 7.84–7.80 (m, 2H), 7.54–7.45 (m, 2H), 7.54–7.45 (m, 6H), 7.21 (d, J = 8.1 Hz, 8H), 7.13 (d, J = 8.1 Hz, 8H), 7.02–6.97 (m, 2H), 4.13–4.10 (m 4H), 2.60 (t, J = 7.7 Hz, 8H), 1.69–1.67 (m, 2H), 1.28 (s, 16H), 1.17–1.15 (m, 32H), 0.88–0.85 (m, 24H).

### 4.3 Preparation of Nanoparticles

The nanoprecipitation method was employed to prepare the NPs. The compound and the amphiphilic polymer F‐127 (Innochem) were thoroughly dissolved in THF to obtain a working solution where the two components were uniformly mixed at a weight ratio of 1:5. Subsequently, under continuous stirring, 1 mL of this working solution was introduced into 9 mL of ultrapure water. To eliminate THF, the mixture was stirred for another 24 h in a fume hood, then filtered through a 220 nm membrane filter to yield a homogeneous nanoparticle (NP) dispersion. The encapsulation yields of NPs are over 70%. The resulting NPs were stored at a constant 4°C in a refrigerator for subsequent use, as described above. The NP sizes were slightly changed after being stored at 4°C for 12 months, suggesting that the structure and size of NPs can keep stable under proper storage conditions (Figure S18).

### 4.4 Electrostatic Potential Calculation

Using Gaussian 09, molecular electrostatic potential (ESP) was computed via DFT at the B3LYP/6‐31G** level, and the B3LYP/6‐31 g (d, p) basis set was employed in Gaussian to optimize the geometry of a single molecule. Dimer clusters with different relative positions were generated using the genmer package within Molclus. The structure of the dimer clusters was optimized using GFN1‐xTB by calling the xtb package via Molclus. The structure of the lowest‐energy cluster from the xtb calculation was further optimized by invoking Gaussian with the M062X/6‐31G* basis set. A virtual atom was introduced at the center of each single molecule in the optimized result. Dimer scanning was used to establish the initial spacing and step size, and coordinate files were generated accordingly. These coordinate files were processed using the xyz2QC package in Molclus, and Gaussian batch files were created. Single‐point energy calculations were performed using Gaussian with the PM6D3 basis set [43, 44].

### 4.5 Determination of Quantum Yield of the NPs

The quantum yield (QY) of the NPs was determined using IR‐1061 (HY‐D1449, MCE) as a reference (QY = 1.7% in dichloromethane), as described below. IR‐1061 was diluted in dichloromethane to prepare a series of standard samples. Photoluminescence (PL) spectra were collected under 808 nm excitation, and the emission spectra were integrated. The same procedure was performed for the samples in water. The integrated emission intensity was plotted against the absorbance, and a linear fit was applied. The QY was calculated based on the following equation:

$$QY_{\mathrm{sample}}=QY_{\mathrm{refer}}\times \frac{K_{\mathrm{sample}}}{K_{\mathrm{refer}}}\times {\left(\frac{n_{\mathrm{sample}}}{n_{\mathrm{refer}}}\right)}^{2}$$

where QY_sample_ is the quantum yield of the three nanoparticles, QY_refer_ is the QY of IR‐1061 as reference, *K*_sample_ and *K*_refer_ are the slopes obtained by linear fitting of the integrated emission spectra of the AIEgens, nanoparticles, and IR‐1061 against the absorbance at 808 nm, *n*_sample_ and *n*_refer_ are the refractive index [45].

### 4.6 Photothermal Properties of NPs

To assess the photothermal effect, nanoparticles at varying concentrations were exposed to an 808 nm laser (0.33 W·cm^−^^2^, 5 min), with the laser power density calibrated via a spectroradiometer. During these procedures, the temperature was recorded at 30‐s intervals using a portable infrared thermometer.

### 4.7 Photostability of NPs

To evaluate photostability, the photothermal response of NP aqueous solutions (100 µg·mL^−^^1^) was monitored over five repeated on/off cycles of 808 nm irradiation (0.33 W·cm^−^^2^, 5 min on, followed by 5 min off for natural cooling). During these procedures, the temperature was recorded at 30‐s intervals using a portable infrared thermometer. ICG (HY‐D0711, Mce) has been used as a standard control for comparison.

### 4.8 Calculation of NPs Photothermal Conversion Efficiency

The specific calculation method can be referred to in this literature [46].

(1) $$PCE=\frac{hS\left(\Delta T_{NPs}-\Delta T_{\mathrm{surr}}\right)-Q_{S}}{I(1-10^{-A_{\lambda }})}$$

In this expression, *PCE* represents the photothermal conversion efficiency, whereas Δ*T*_NPs_ and Δ*T*_surr_ denote the equilibrium temperature of the NPs (°C) and the ambient temperature (°C), respectively. *Q_S_* corresponds to the baseline energy supplied by the sample cell, *I* refers to the 808 nm laser power (mW), and *A*_λ_ indicates the NP absorbance at 808 nm.

Within this equation, only *hS* remains undetermined, which denotes the dimensionless driving force temperature (mW). *h* and *S* stand for the heat transfer coefficient and the container's surface area, respectively. To determine *hS*, we introduce θ, defined as the ratio of Δ*T* to Δ*T*_max _:

(2) $$\theta =\frac{\Delta T}{\Delta T_{\max}}=\frac{T-T_{\min}}{T_{\max}-T_{\min}}$$

where *T* is the solution temperature.

(3) $$t=-\frac{\sum_{i}m_{i}C_{p,i}}{hS}ln\theta$$

Let *t* denote the cooling time points after 5 min of continuous irradiation, *m* the water mass (g), and *C_p_* the heat capacity of water (4.2 J/g). The value of *C_p_* can be obtained from the linear relationship between time and − *ln*θ. Compared with the solvent (water, 1 × 10^−^^3^ kg), the NP mass is negligible. Because water's specific heat greatly exceeds that of most materials, the contributions of *m*_*NP*s_ and *C*_*p*,*NPs*_ from the NPs are ignored. Returning to equation (a), all parameters are now determined.

The Δ*T*_NPs_ of IEICO‐F, IEICO‐Cl, and IEICO are respectively 35.3, 30.9, 26.1°C; the Δ*T*_surr_ is 0.3°C; the *I* is 400 mW; the light spot is 1.212 cm^2^. The*A*_λ_of IEICO‐F, IEICO‐Cl, and IEICO are 0.478, 0.31, 0.287, respectively. The *hS* are calculated by formula (c) and are respectively 5.00 × 10^−3^, 4.13 × 10^−3^, 4.26 × 10^−3^. Therefore, the photothermal conversion efficiency (PCE) of IEICO‐F, IEICO‐Cl, and IEICO NPs can be calculated as 65.59%, 61.88%, 56.76%, respectively.

### 4.9 Cell Culture

143B, KHOS, and K7M2 cells were derived from ATCC, and HUVECs were supplied by Wuhan Pricella Biotechnology Co., Ltd. All cell samples underwent STR authentication and tested negative for mycoplasma infection. Cell cultivation was maintained in DMEM (C11995500BT, Gibco) containing 10% fetal bovine serum (086 150, Wisent) as well as 1% penicillin/streptomycin (15140 122, Gibco). Phosphate buffer saline (C10010500BT, Gibco) was applied for cell rinsing, and 0.25% Trypsin EDTA (25200 072, Gibco) was used for cell dissociation. Cell culture incubation was sustained at 37°C within humidified air with 5% CO_2_. For long‐term cryopreservation, cell samples were preserved in freezing medium (C40100, New Cell & Molecular Biotech) under −80°C environment. Every experimental reagent was handled strictly in accordance with official manufacturer protocols.

### 4.10 CCK‐8 Assay

143B, KHOS, HUVEC, and K7M2 cells were seeded into 96‐well plates at a density of 1 × 10^4^ cells per well in 100 µL of medium. After 24 h of incubation, cells were treated with nanoparticles (NPs) at various concentrations (0, 12.5, 25, 50, 100, 200 µg·mL^−^^1^) for 24 or 48 h to assess NP biosafety. Each concentration and the control group were set up with three replicate wells to ensure data accuracy. For in vitro photothermal therapeutic cytotoxicity assessment, cells received 808 nm laser stimulation at 0.33 W·cm^−^^2^ for 5 min under dark surroundings, followed by another 6 h of sustained cultivation. The old culture medium was then discarded from 96‐well plates, and 100 µL fresh full medium supplemented with 10% CCK‐8 reagent (CK04, Dojindo) was replenished for 2 h of continuous incubation. Optical absorbance at 450 nm was ultimately detected via a Biotek microplate reader, while blank cells cultured in pure medium served as the negative reference group.

### 4.11 Live/Dead Cell Staining

143B and KHOS cells were plated into 24‐well chambers at a density of 5 × 10^4^ cells per well and cultured overnight at 37°C. Initial culture liquids were discarded and replaced with fresh DMEM supplemented with 100 µg/mL nanoparticle formulations. After 24 h of continuous cultivation, cellular samples underwent 808 nm laser treatment (0.33 W·cm^−^^2^, 5 min) under dark conditions. Live‑dead dual staining was conducted using Calcein AM and propidium iodide (PI, Aladdin), and fluorescent imaging was lastly adopted to distinguish viable and damaged cell populations.

### 4.12 Animal Experiments and Tumor Models

All in vivo operations gained ethical authorization from the Ethics Committee of Peking University People's Hospital (Approval ID: 2024PHE030). Six‐week‐old BALB/c nude mice from Beijing Vital River were applied to construct subcutaneous tumor xenografts. All rodents were anesthetized via isoflurane inhalation, and their right dorsal regions were disinfected using 75% ethyl alcohol. A total of 5 × 10^6^ 143B cells suspended in 100 µL serum‐free medium were injected subcutaneously into each mouse. When tumor bulk grew over 100 mm^3^, laboratory animals were randomized into separate cohorts with comparable tumor sizes. Individual tumor growth and body weight were documented every third day, and tumor size was quantified through the formula: length × width^2^ / 2.

### 4.13 In Vivo Tumor Eradication via Photothermal Action

Tumor‐bearing 143B xenograft mice were randomized into four distinct cohorts: PBS, PBS plus laser, IEICO‐F NPs alone, and combined NPs with laser. Animals in laser‐treated groups received tail vein delivery of 100 µL PBS or 200 µg/mL IEICO‐F nanoparticle dispersions for subsequent photothermal management. At 24 h post‐injection, tumor sites were irradiated with an 808 nm laser for 5 min. As controls, the PBS and NPs groups received the same injections but without laser irradiation. Temperature changes were recorded using an infrared thermal camera.

### 4.14 Liver and Kidney Function Parameters and H&E Staining

Twenty‐four hours after tail vein injection of PBS or IEICO‐F NPs, mouse serum was collected, and levels of ALT, AST, CR, TBIL, and UREA were detected using a fully automated biochemical analyzer (BS‐240Vet). On the 15th experimental day, all experimental rodents were humanely sacrificed. Key visceral tissues including heart, liver, spleen, lung, and kidney were harvested, stabilized in 4% paraformaldehyde, embedded in paraffin, sliced into thin sections, and processed for routine H&E histological staining.

### 4.15 Statistical Analysis

Quantitative results are expressed as mean values with standard deviation. Unpaired two‐tailed t‐tests assessed differences between two independent cohorts, and one‐way ANOVA analyzed multigroup datasets. All statistical calculations were completed with GraphPad Prism 9.0, whereas Origin 2019 and Prism software jointly generated all experimental graphs. Statistical significance was defined as *p < 0.05, **p < 0.01, ***p < 0.001, and ****p < 0.0001, with ns indicating no obvious statistical difference.

## Conflicts of Interest

The authors declare no conflicts of interest.

## Supporting information




**Supporting File**: advs75482‐sup‐0001‐SuppMat.docx.

## Data Availability

The data that support the findings of this study are available on request from the corresponding author. The data are not publicly available due to privacy or ethical restrictions.
