# Supplementary material for: A Novel Approach Based on Acceptor–Donor–Acceptor Type Molecules Enhances the Therapeutic Efficacy and Safety of Photothermal Therapy for Osteosarcoma
Source: Adv Sci (Weinh). 2026 May 14;13(42):e75482. doi: 10.1002/advs.75482 (PMC13335441; doi:10.1002/advs.75482)
Supplement: Supplementary file 1 — Supporting File: advs75482‐sup‐0001‐SuppMat.docx. [file ADVS-13-e75482-s001.docx]

**Supplementary Manuscript**

**A Novel Approach Based on Acceptor-Donor-Acceptor Type Molecules Enhances the Therapeutic Efficacy and Safety of Photothermal Therapy for Osteosarcoma**

Zhijian Jin^1,2,#^, Hongru Wang^3,#^,Yi Yang^1,2,#^, Yuhang Xie^4^, Yuan Zhang^1,4^, Zhiye Du^1,2^, Yi Huang^1,2^, Wei Guo^1,2,*^, Yang Li^5,*^, Shidong Wang^1,2,*^

1. Department of Musculoskeletal Tumor, Peking University People**’**s Hospital, Beijing, 100044, China

2. Beijing Key Laboratory of Musculoskeletal Tumor, Peking University People**’**s Hospital, Beijing, 100044, China

3. Department of Neurology, Liaocheng People's Hospital, Liaocheng, Shandong, 252000, China

4. Peking University Health Science Center, Beijing, 100080, China

5. Department of General Surgery, the First Medical Center, Chinese PLA General Hospital, Beijing, 100039, China

**Co-first authors^#^**

Zhijian Jin, Hongru Wang, and Yi Yang contributed equally to this work and should be considered as co-first authors.

**Corresponding Authors***

Shidong Wang (Lead contact：[stonewang@bjmu.edu.cn](mailto:stonewang@bjmu.edu.cn)), Yang Li ([421758116@qq.com](mailto:421758116@qq.com)), and Wei Guo ([bonetumor@163.com](mailto:bonetumor@163.com)).

**Competing Interests**

The authors declare no potential conflicts of interest.


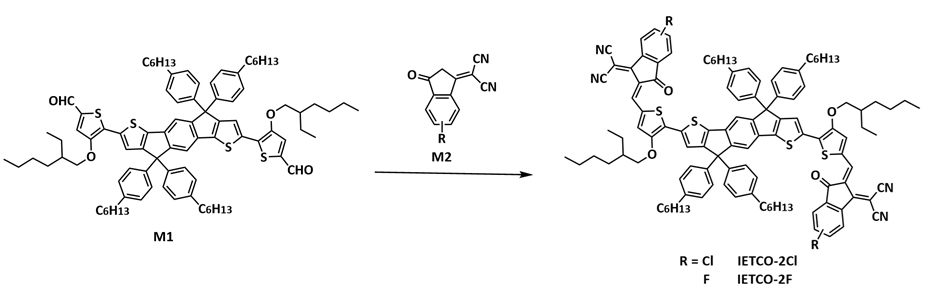


**Figure S1**. Synthesis of IEICO-F/Cl.


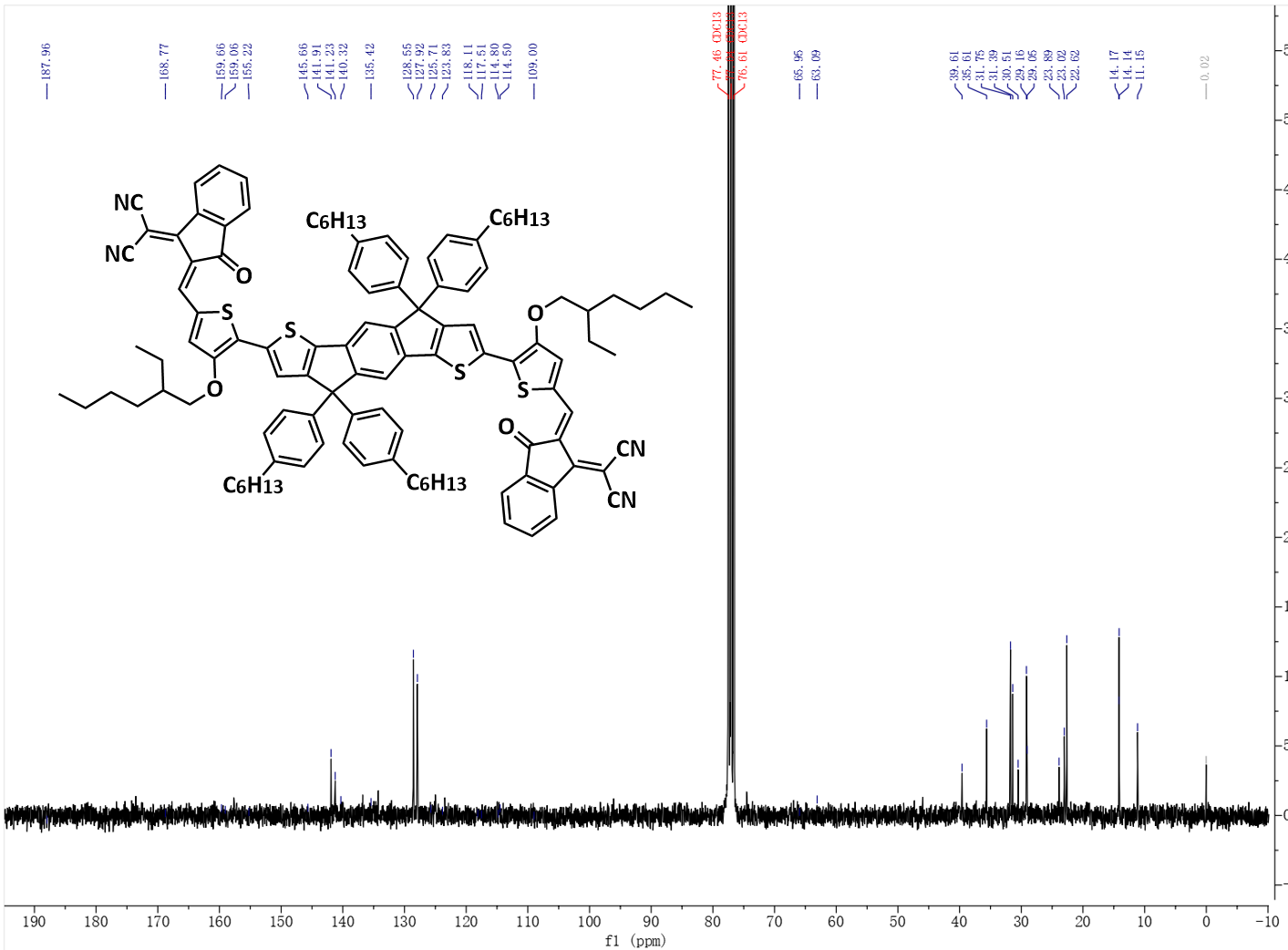


**Figure S2.** ^13^C-NMR spectrum (400 MHz) of IEICO.


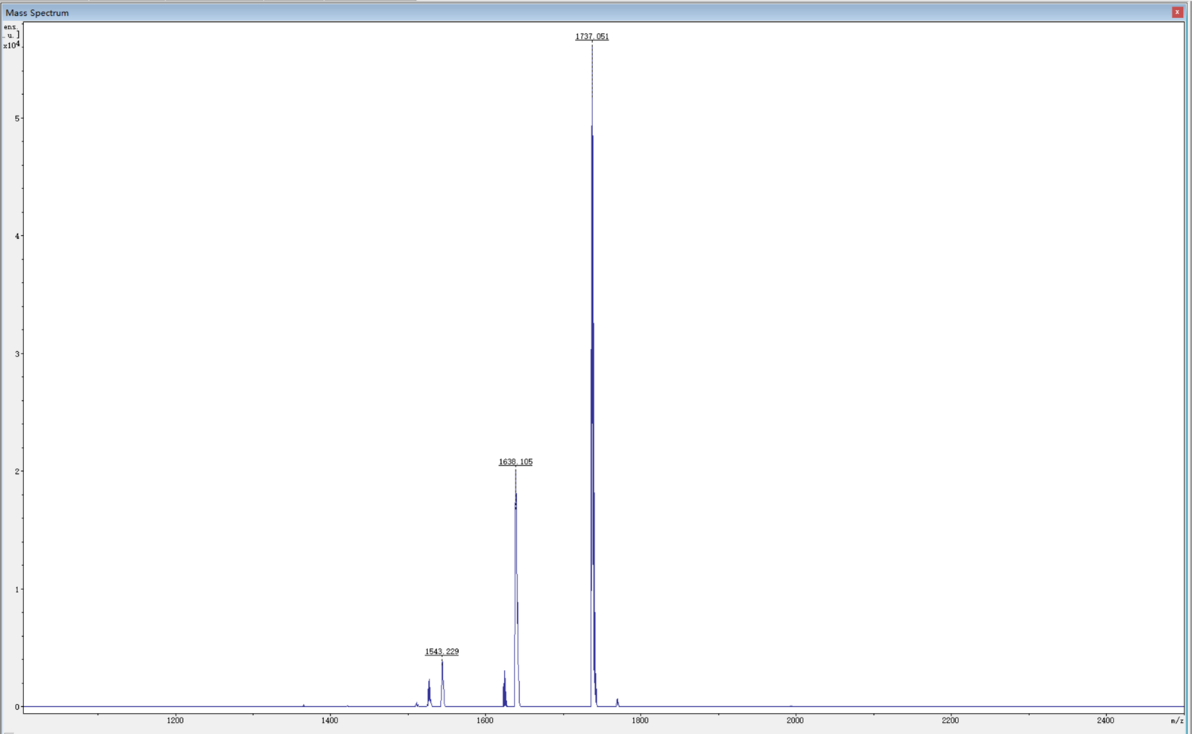


**Figure S3.** The MALDI-TOF spectrum of IEICO.

IEICO ^13^C NMR (400 MHz, CDCl3, δ): 187,95, 168,77, 159,66, 155.22, 154,70, 145.66, 141.91, 141.23, 140.32, 135.43, 128.55, 127.92, 125.71, 123.83, 118.11, 117.51, 114.80, 114.50, 109.00, 76.51, 65.95, 63.09, 39.61, 35.61, 31.39, 30.51, 29.16, 29.05, 23.89, 23.02, 22.62, 14.17, 14.14, 11.15. HRMS (MALDI-TOF, m/z) Calcd for C_114_H_118_N_4_O_4_S_4_ [M^+^]: 1737.80. Found: 1737.05.


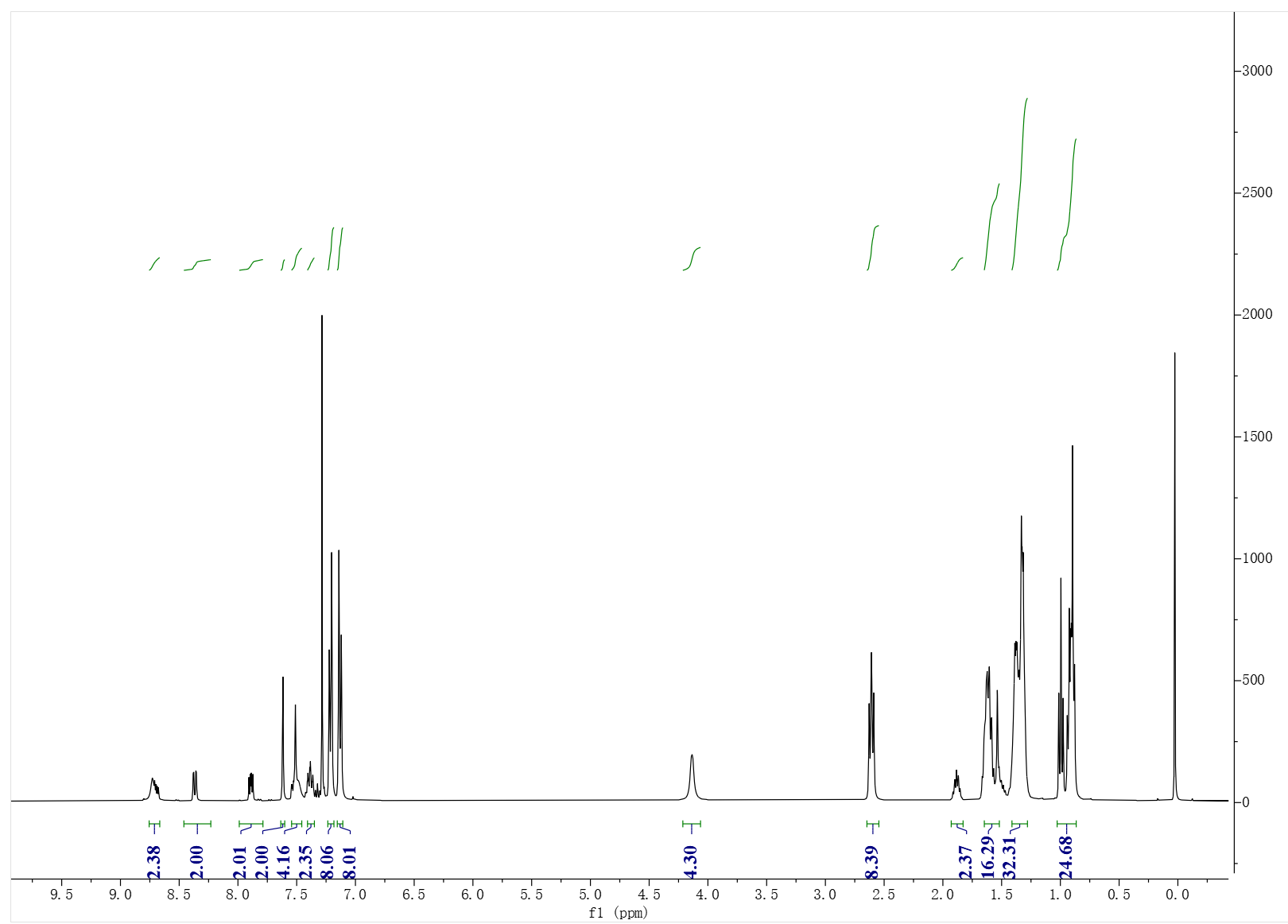


**Figure S4.** ^1^H-NMR spectrum (400 MHz) of IEICO-F.


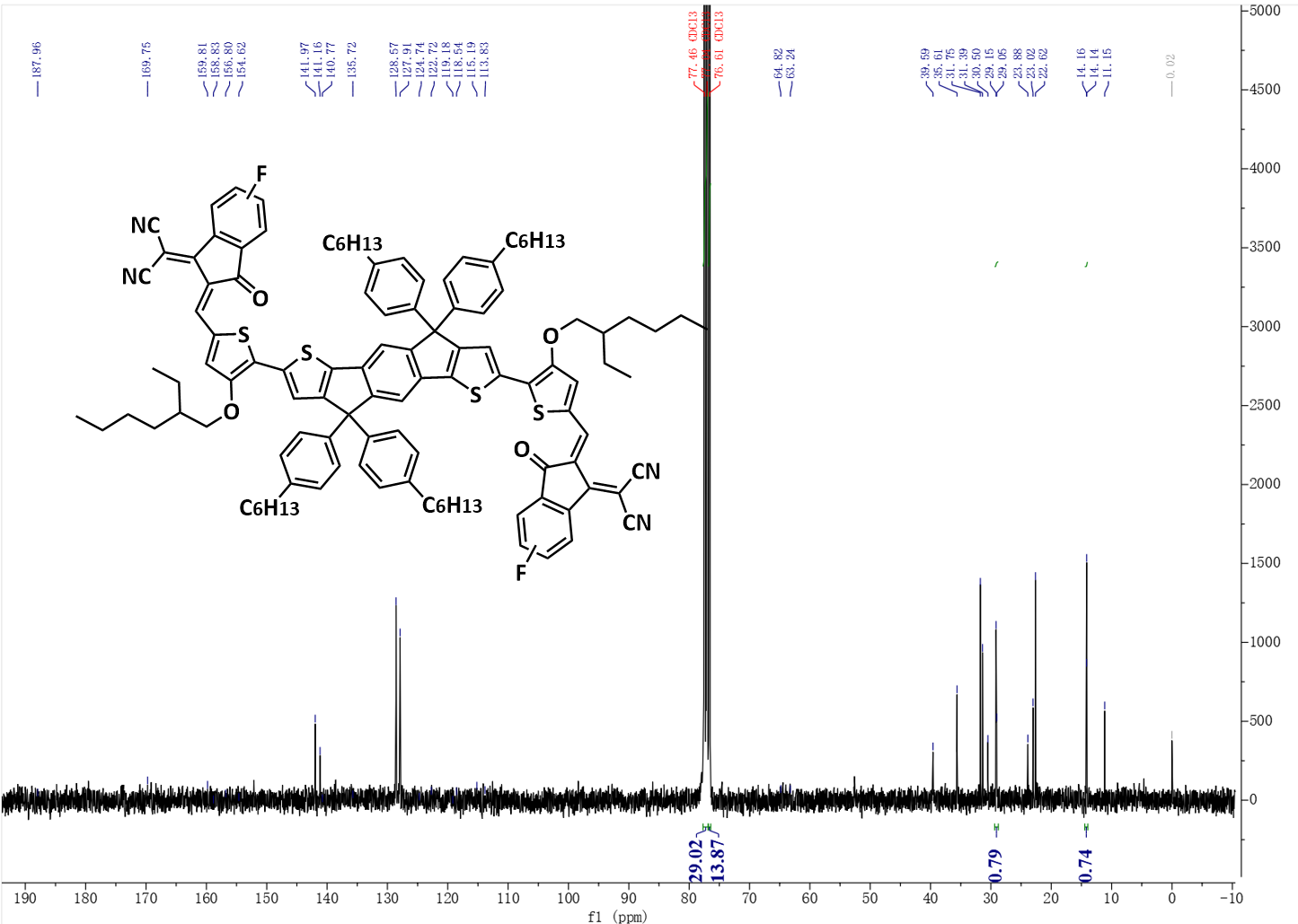


**Figure S5.** ^13^C-NMR spectrum (400 MHz) of IEICO-F.


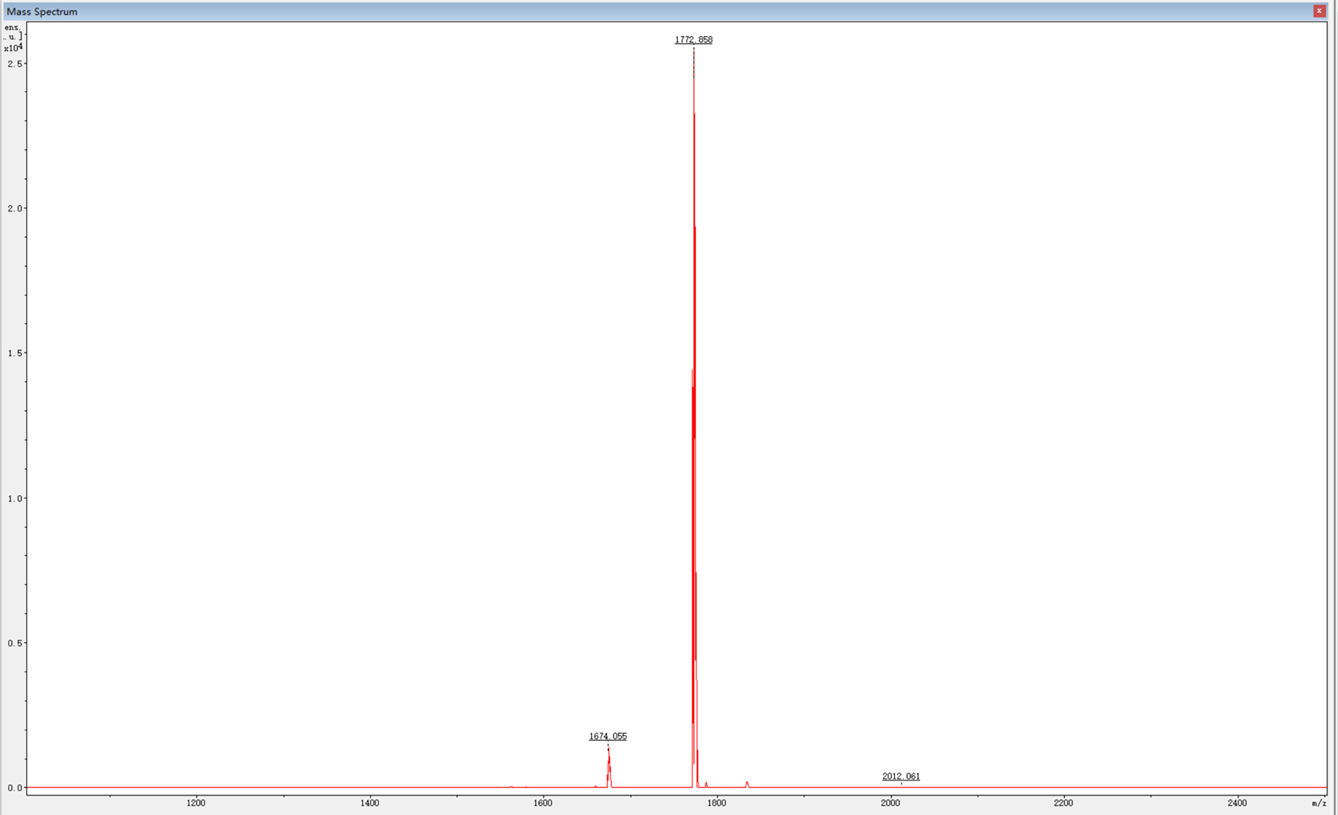
 **Figure S6.** The MALDI-TOF spectrum of IEICO-F.

IEICO-2F ^1^H NMR (400 MHz, CDCl3, δ) 8.75–8.63 (m, 2H), 8.37 (dd, J = 9.0, 2.1 Hz, 2H), 7.89 (dd, J = 8.3, 5.1 Hz, 2H), 7.62 (d, J = 2.4 Hz, 2H), 7.53 (d, J = 13.2 Hz, 4H), 7.39 (tt, J = 5.9, 2.8 Hz, 2H), 7.21 (d, J = 8.0 Hz, 8H), 7.13 (d, J = 8.0 Hz, 8H), 4.13 (s, 4H), 2.61 (t, J = 7.8 Hz, 8H), 1.88 (p, J = 6.0 Hz, 2H), 1.65–1.53 (m, 16H), 1.42–1.29 (m, 32H), 1.02 – 0.86 (m, 24H).^13^C NMR (400 MHz, CDCl3, δ): 187,95, 169.75, 159,81, 158.83, 156.80, 154.62, 145.66, 141.97, 141.16, 140.77, 135.72, 128.57, 127.91, 124.74, 122.72, 119.81, 118.54, 115.19, 113.83, 76.51, 64.82, 63.24, 39.59, 35.61, 31.75, 30.50, 29.15, 29.05, 23.88, 23.02, 22.62, 14.16, 14.14, 11.15. HRMS (MALDI-TOF, m/z) Calcd for C_114_H_116_N_4_O_4_S_4_F_2_ [M^+^]: 1772.79. Found: 1772.85.

**
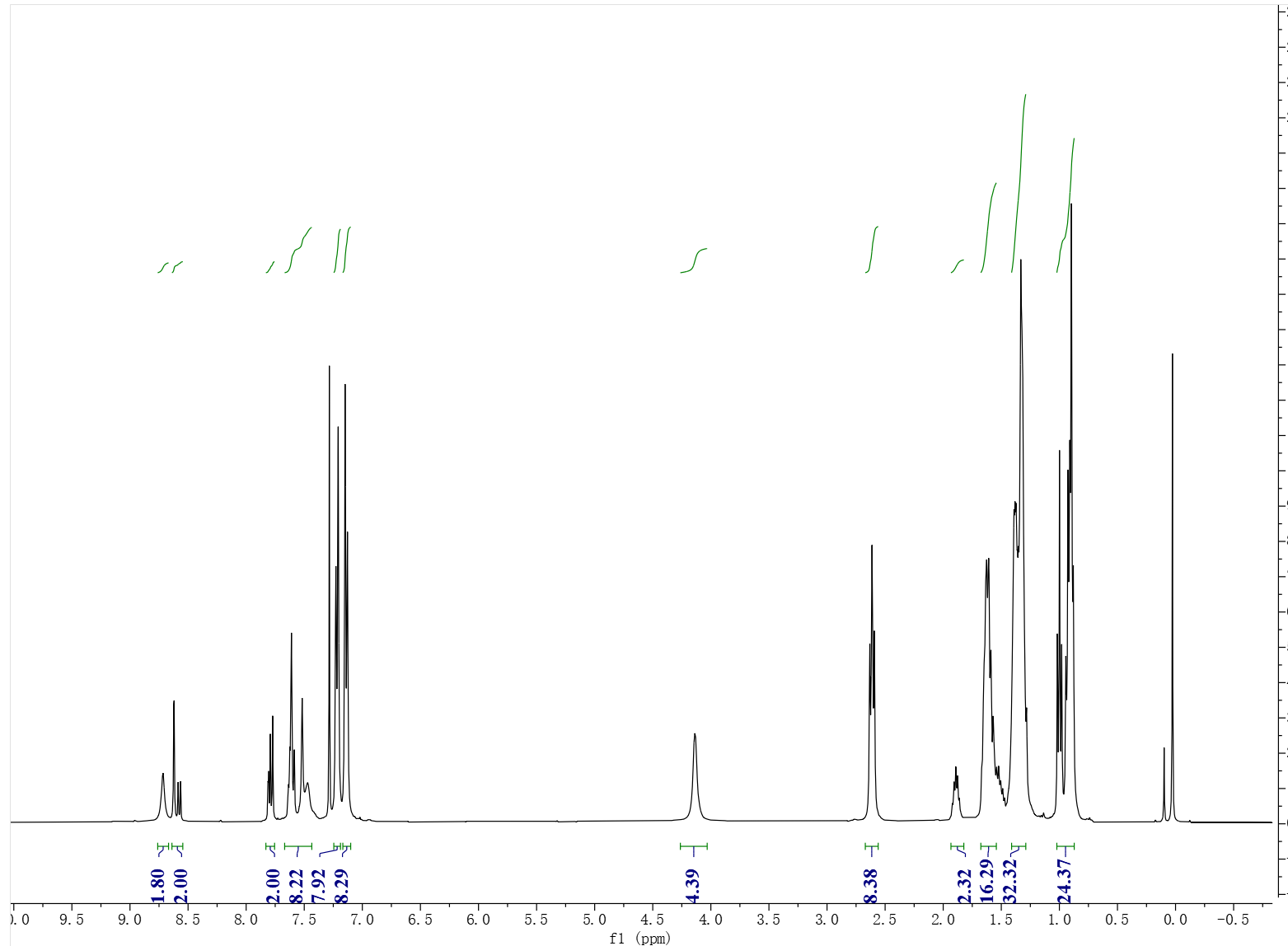
**

**Figure S7.** ^1^H-NMR spectrum (400 MHz) of IEICO-Cl.


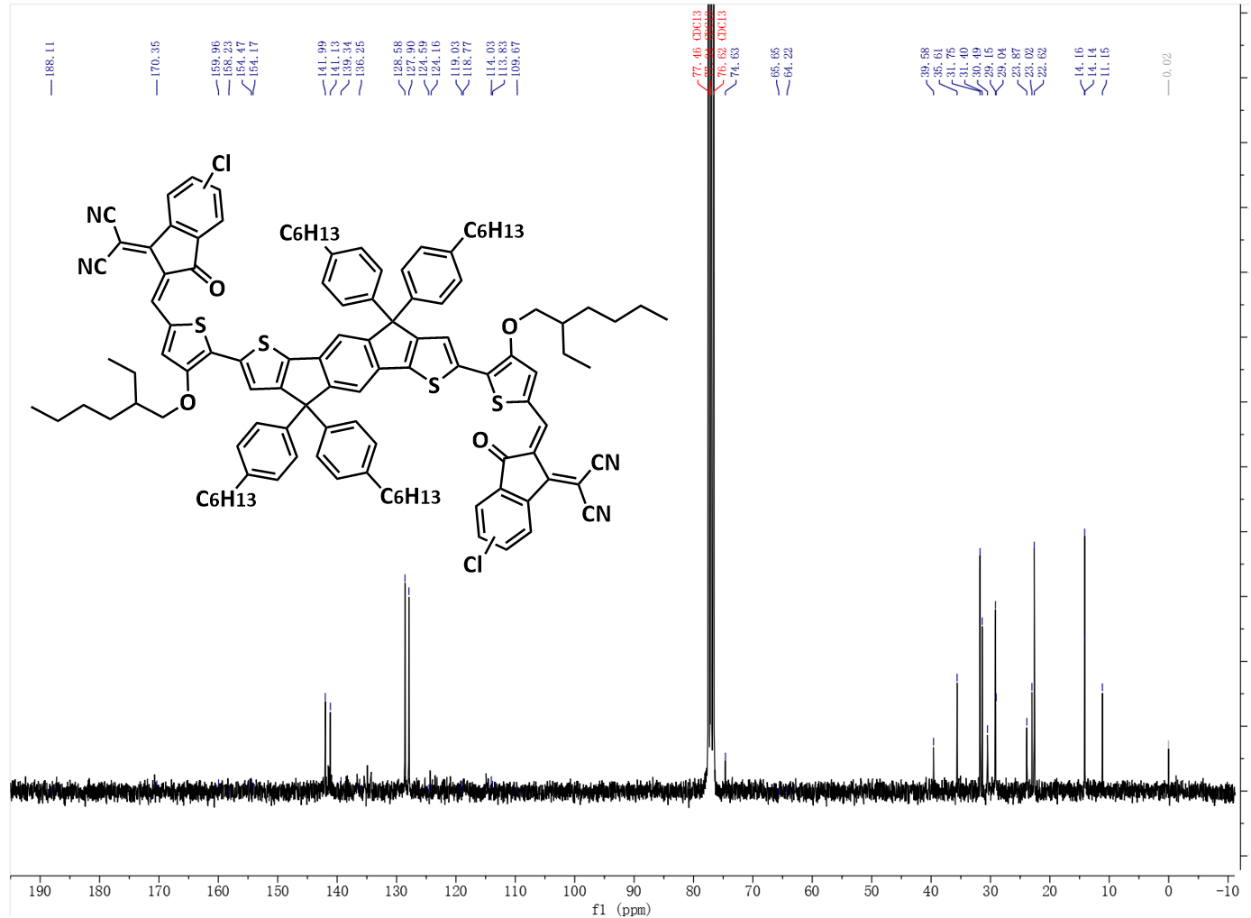


**Figure S8.** ^13^C-NMR spectrum (400 MHz) of IEICO-Cl.


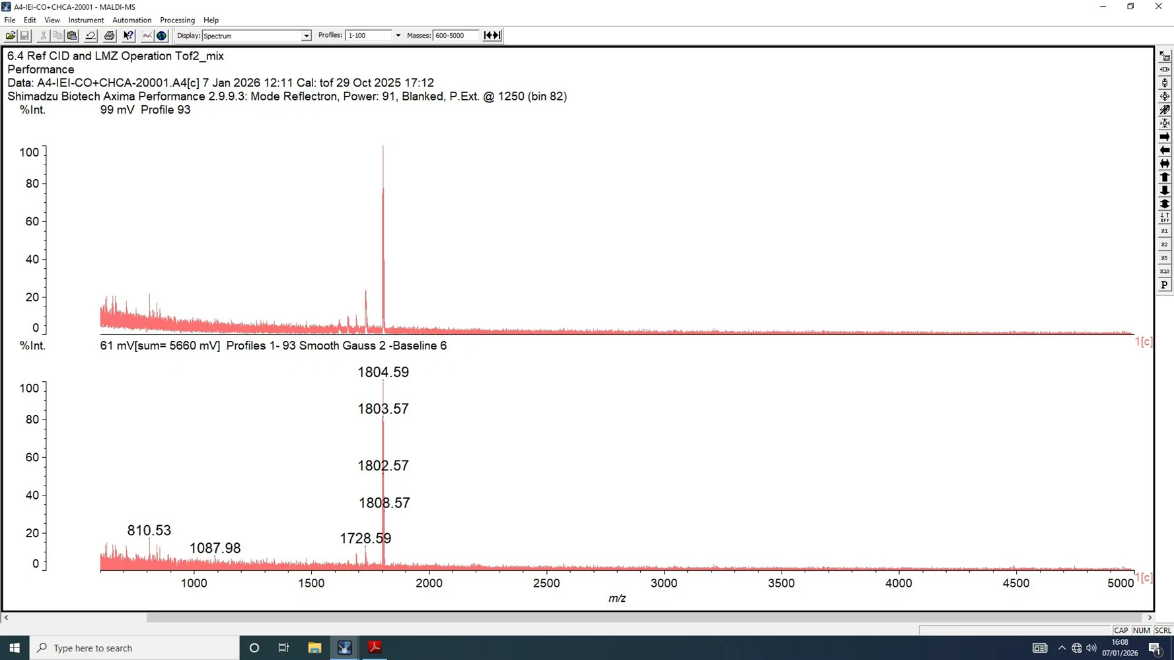


**Figure S9.** The MALDI-TOF spectrum of IEICO-Cl.

IEICO-2Cl ^1^H NMR (400 MHz, Chloroform-d) δ 8.72 (s, 2H), 8.64 – 8.55 (m, 2H), 7.83 – 7.76 (m, 2H), 7.67 – 7.43 (m, 8H), 7.25 – 7.19 (m, 8H), 7.14 (d, J = 7.9 Hz, 8H), 4.14 (s, 4H), 2.61 (t, J = 7.8 Hz, 8H), 1.89 (p, J = 6.1 Hz, 2H), 1.61 (dq, J = 15.3, 7.5 Hz, 16H), 1.41 – 1.29 (m, 32H), 1.02 – 0.87 (m, 24H). ^13^C NMR (400 MHz, CDCl3, δ): 188.11, 170.35, 159,96, 158.22, 154,47, 154.71, 141.99, 141.13, 139,34, 136.25, 128.58, 127.90, 124.59, 124.16, 119.03, 118.77, 114.03, 113.83, 109.67, 74.63, 65.65, 64.22, 39.58, 35.61, 31.75, 30.49, 29.15, 29.04, 23.87, 23.02, 22.62, 14.16, 14.14, 11.15. HRMS (MALDI-TOF, m/z) Calcd for C_114_H_116_N_4_O_4_S_4_Cl_2_ [M^+^]: 1804.74. Found: 17804.59.


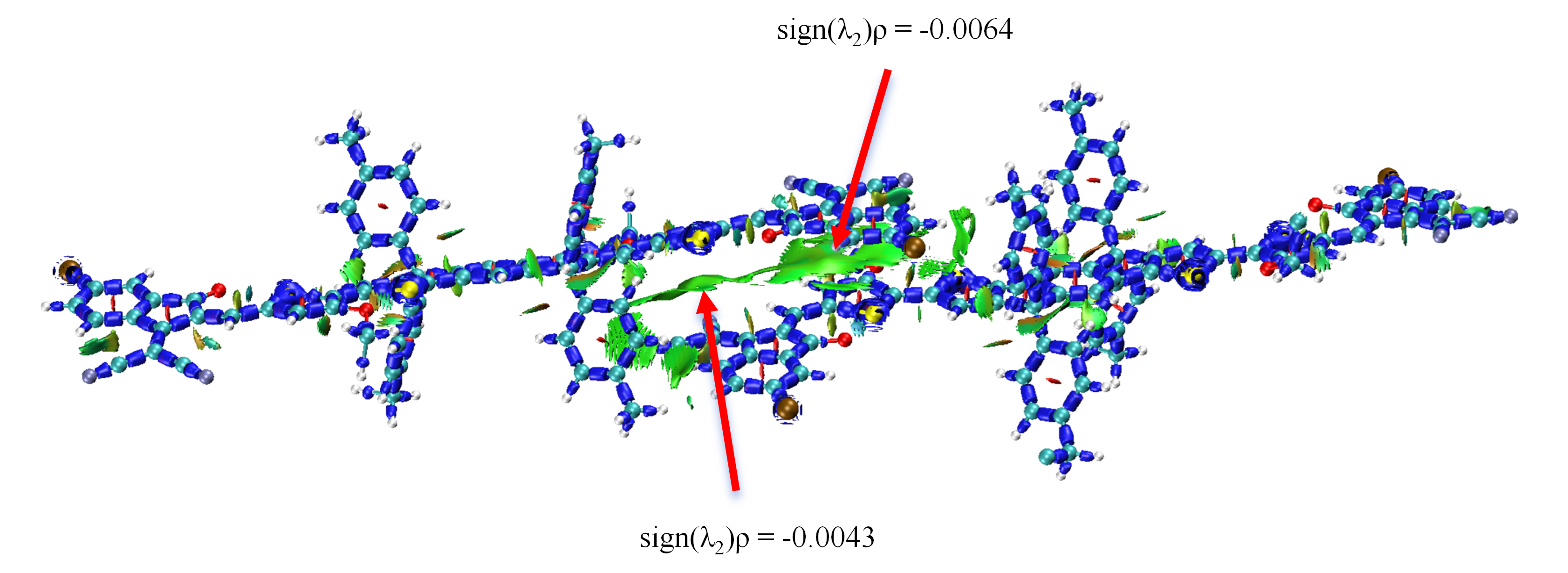


**Figure S10.** NCI maps of two layers for IEICO-Cl.


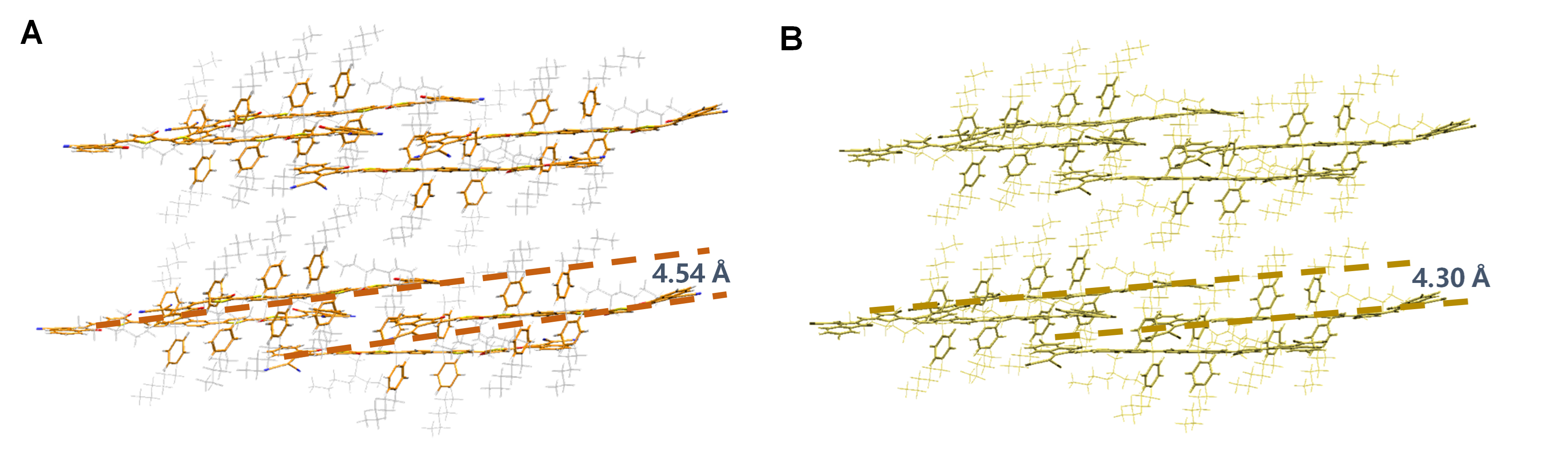


**Figure S11.** Simulation of intermolecular stacking of optimized A) IEICO and B) IEICO-Cl.


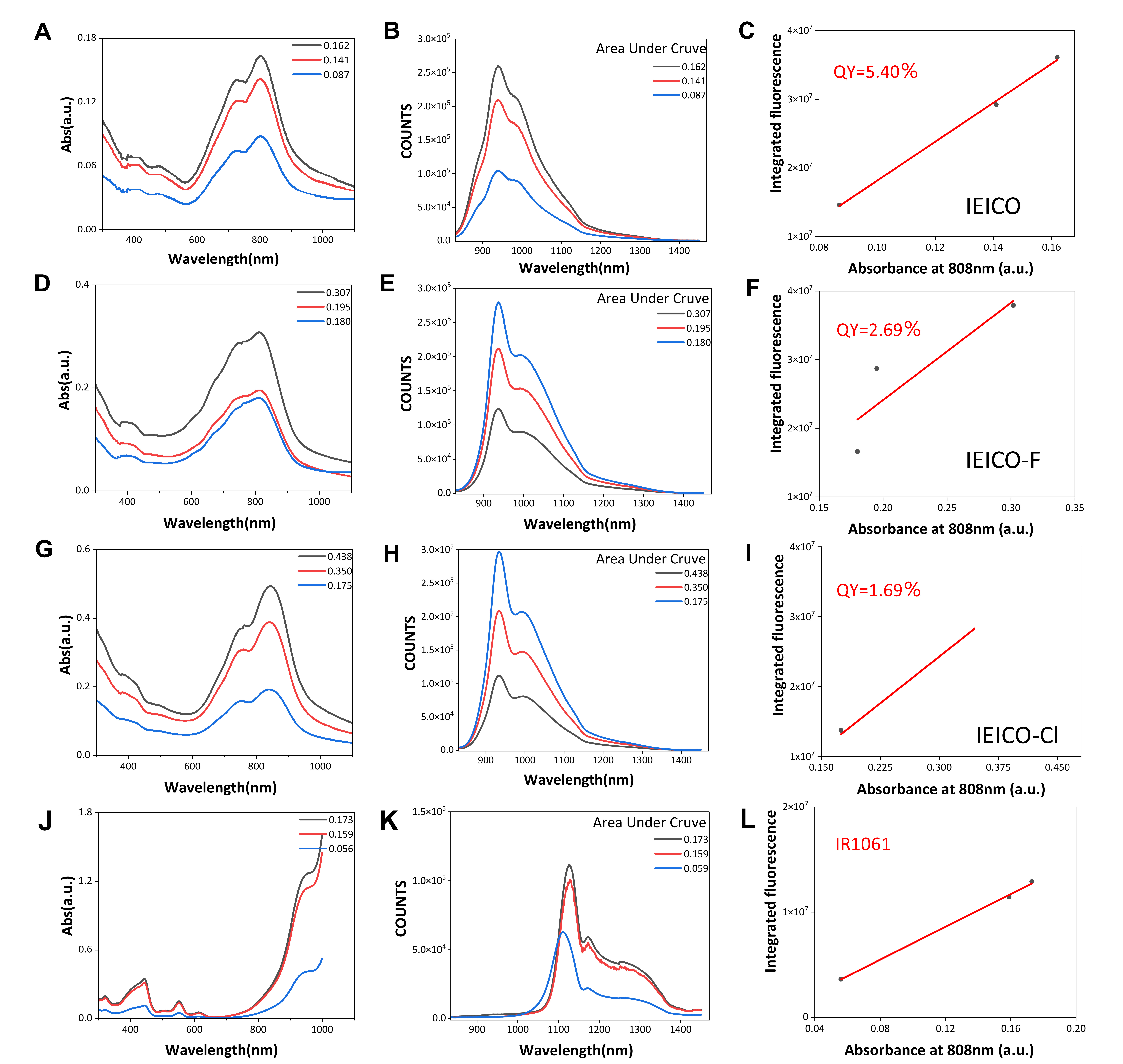


**Figure S12.** UV-vis-NIR absorption spectra of A) IEICO, D) IEICO-F, G) IEICO-Cl solutions, and J) IR-1061 in 1,2-dichloroethane with different concentrations. B, E, H, and K) The emission spectra of all solutions under 808 nm excitation. C, F, I and L) For all solutions, their absorbance values at 808 nm were plotted versus area under curve (AUC) in the emission spectra and fitted into a linear relationship.


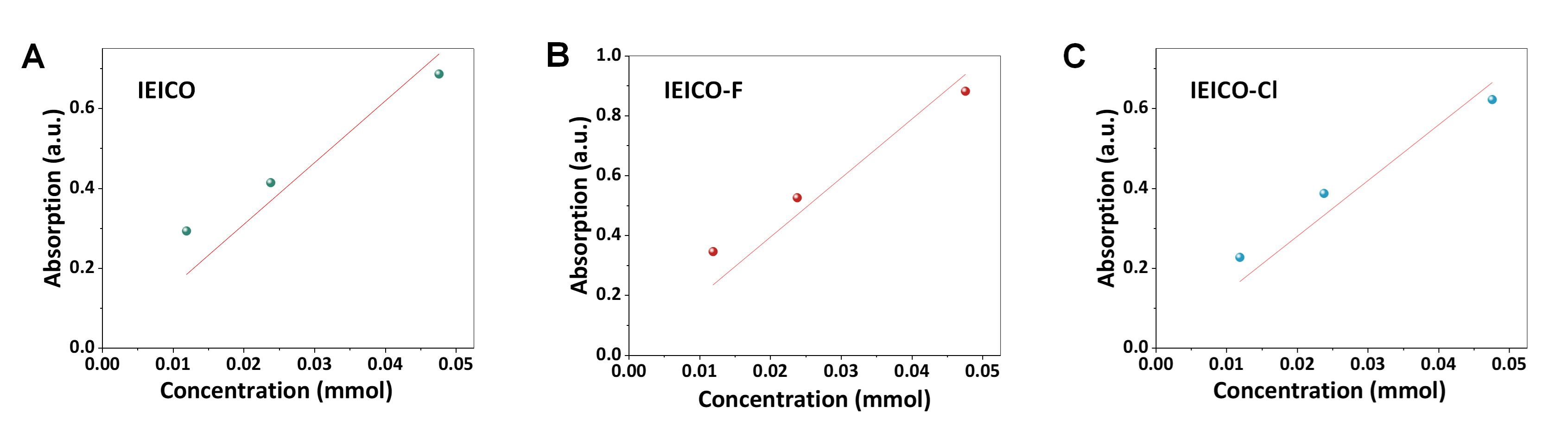
**Figure S13.** Measurement of the mole extinction coefficient of A) IEICO, B) IEICO-F, and C) IEICO-Cl in tetrahydrofuran solutions. The slope of the fitted curve is the mole extinction coefficient of photosensitizers.


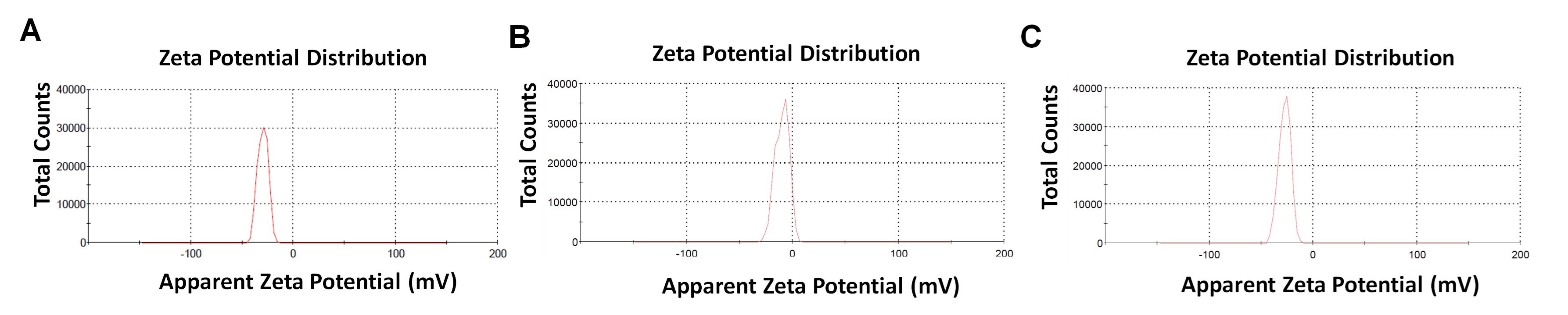


**Figure S14.** Zeta potentials of A) IEICO, B) IEICO-F, and C) IEICO-Cl.


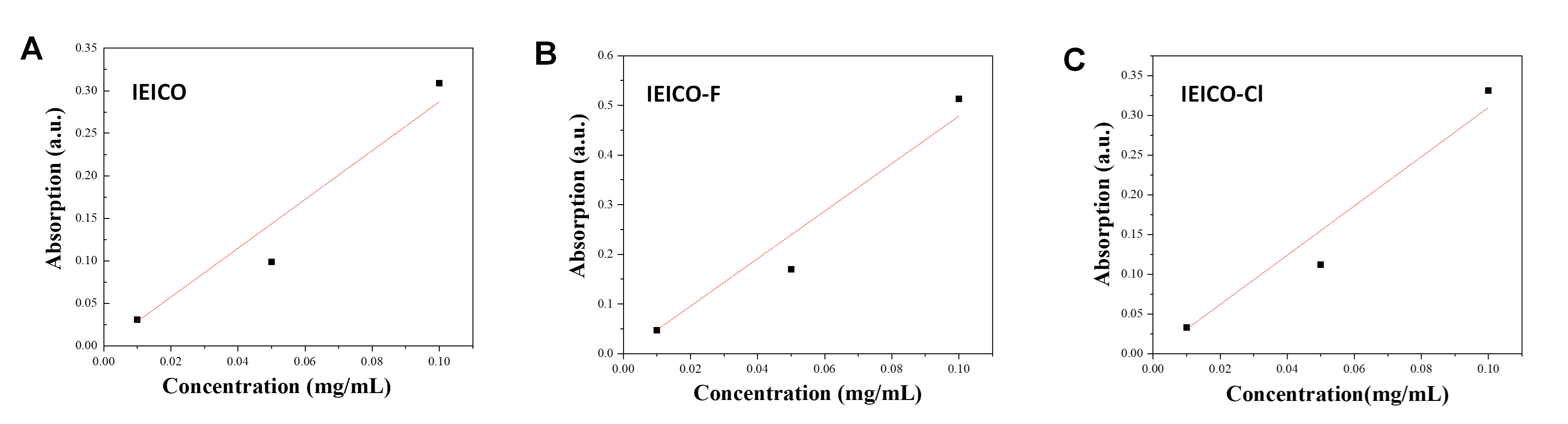


**Figure S15.** Measurement of the mass extinction coefficient of A) IEICO, B) IEICO-F, and C) IEICO-Cl NPs in water. The slope of the fitted curve is the mass extinction coefficient of NPs in water at 808nm.


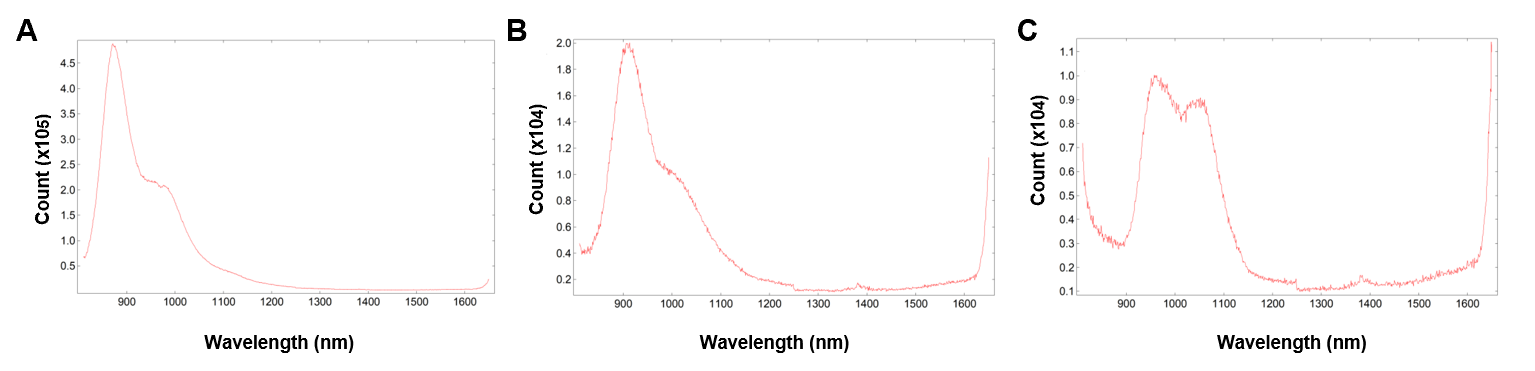


**Figure S16.** The fluorescence emission of A) IEICO, B) IEICO-F, and C) IEICO-Cl NPs in aqueous dispersion.





**Figure S17.** The good biocompatibility and phototherapeutic effect of NPs at the cellular level. Viabilities of IEICO-Cl NPs against cancerous A) 143B, B) KHOS, and noncancerous C) HUVEC cells after 24 and 48 h of incubation at different concentrations. Viabilities of IEICO NPs against cancerous D) 143B, E) KHOS, and noncancerous F) HUVEC cells after 24 and 48 h of incubation at different concentrations. Viabilities of cancerous G) 143B, H) KHOS, and I) K7M2 cells incubated with IEICO-Cl NPs with or without laser irradiation (808 nm, 0.33 W·cm^-2^, 5 mins). Viabilities of cancerous J) 143B, K) KHOS, and L) K7M2 cells incubated with IEICO NPs with or without laser irradiation (808 nm, 0.33 W·cm^-2^, 5 mins). Data are presented as the mean ± SD, n = 3. *p < 0.05, **p < 0.01, ***p < 0.001, ****p < 0.0001, ns, no significance. NPs, nanoparticles.


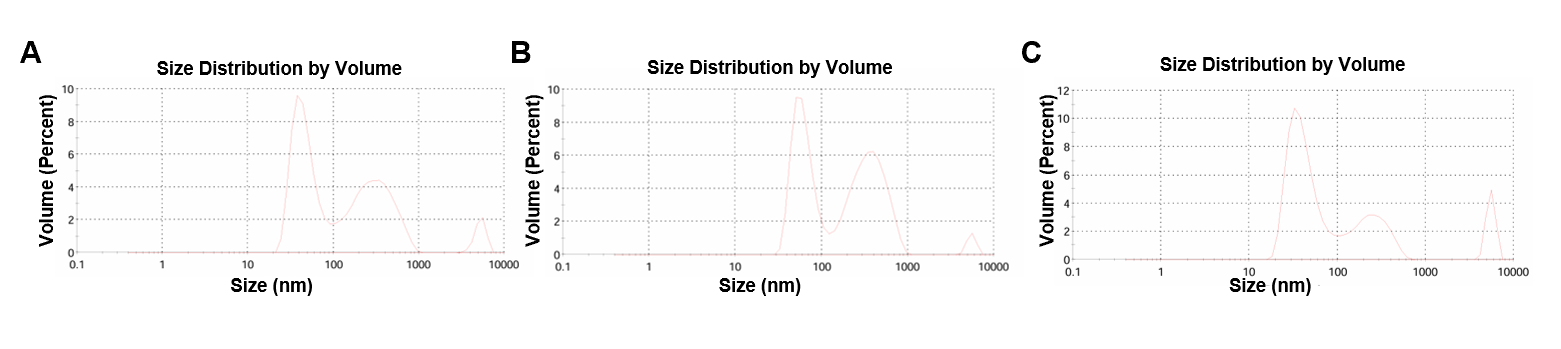
 **Figure S18.** Dynamic light scattering profile of A) IEICO, B) IEICO-F, and C) IEICO-Cl NPs.

**Table S1.** Weight data of mice during treatment

| Group | Time/Day | Mouse 1  Weight (g) | Mouse 2  Weight (g) | Mouse 3  Weight (g) | Mouse 4  Weight (g) | Mouse 5  Weight (g) | Average weight (g) |
| --- | --- | --- | --- | --- | --- | --- | --- |
| PBS | 0  3  6  9  12  15 | 25.5  27.6  28.5  29.9  30.3  31.3 | 24.9  25.5  26.3  27.5  28.5  29.0 | 23.5  24.1  23.7  24.9  26.3  25.7 | 26.9  27.0  27.4  28.8  27.1  30.5 | 29  28.9  28.7  29.7  30.4  31.4 | 25.96  26.62  26.92  28.16  28.52  29.58 |
| PBS+Laser | 0  3  6  9  12  15 | 26.4  27.2  27.8  29.0  30.4  31.7 | 23.0  24.1  24.5  24.9  25.0  25.3 | 26.7  26.8  26.9  28.4  29.7  30.7 | 26.2  26.4  26.1  27.6  28.1  29.4 | 25.4  25.9  26.2  27.2  27.9  28.7 | 25.54  26.08  26.30  27.42  28.22  29.12 |
| NPs | 0  3  6  9  12  15 | 24.8  25.9  26.5  28.0  29.6  30.6 | 25.1  25.3  25.0  25.8  27.0  27.8 | 25.1  24.4  24.9  26.0  27.3  28.5 | 24.3  24.6  24.5  25.8  26.4  27.6 | 27.1  26.1  27.4  28.3  29.7  31.0 | 25.28  25.26  25.66  26.78  28.00  29.10 |
| NPs+Laser | 0  3  6  9  12  15 | 24.8  26.0  26.2  27.1  27.5  28.1 | 25.0  25.4  25.7  26.8  27.2  28.5 | 25.7  26.8  26.6  28.0  26.9  27.7 | 24.7  24.0  24.0  25.3  27.9  28.2 | 26.5  26.3  26.5  27.9  27.8  28.7 | 25.34  25.70  25.80  27.02  27.46  28.24 |
